# Automated glycan assembly of arabinomannan oligosaccharides from *Mycobacterium tuberculosis*

**DOI:** 10.3762/bjoc.15.288

**Published:** 2019-12-06

**Authors:** Alonso Pardo-Vargas, Priya Bharate, Martina Delbianco, Peter H Seeberger

**Affiliations:** 1 Department of Biomolecular Systems, Max-Planck-Institute of Colloids and Interfaces, Am Mühlenberg 1, 14476 Potsdam, Germany; 2 Institute of Chemistry and Biochemistry, Freie Universität Berlin, Arnimallee 22, 14195 Berlin, Germany

**Keywords:** arabinomannan, automated glycan assembly, capping

## Abstract

Arabinomannan (AM) polysaccharides are clinical biomarkers for *Mycobacterium tuberculosis* (MTB) infections due to their roles in the interaction with host cells and interference with macrophage activation. Collections of defined AM oligosaccharides can help to improve the understanding of these polysaccharides and the development of novel therapeutical and diagnostic agents. Automated glycan assembly (AGA) was employed to prepare the core structure of AM from MTB, containing α-(1,6)-Man, α-(1,5)-Ara, and α-(1,2)-Man linkages. The introduction of a capping step after each glycosylation and further optimized reaction conditions allowed for the synthesis of a series of oligosaccharides, ranging from hexa- to branched dodecasaccharides.

## Introduction

Bacterial infections caused by MTB killed 1.7 million people in 2017. Additionally, more than 10 million new tuberculosis (TB) cases were reported, with multidrug-resistant TB accounting for almost 10% of the registered cases [1]. The development of novel therapeutic agents and more efficient strategies to detect MTB infections at an early stage is essential, as an early diagnosis would help to prevent most deaths from tuberculosis [1]. AMs, one of the main components of the mycobacterial cell wall [2], are composed of α-(1,5)-, α-(1,3)- and β-(1,2)-arabinoses as well as α-(1,6)- and α-(1,2)-mannosides [3–5]. AMs are potential clinical biomarkers for infection [6–10] due to their roles in the interaction with host cells, interference with macrophage activation, and immunosuppression of T cell responses [11–12]. Diagnosis of tuberculosis in patients with HIV coinfections is possible by immunodetection of AMs and their lipidated lipoarabinomannan (LAM) analogs in urine samples using the Alere lateral flow urine (LF)-LAM assay [13]. However, the low sensitivity of the Alere assay limits its scope. Glycan array screening of more than 100 monoclonal antibodies against more than 60 synthetic glycans related to the mycobacterial cell wall helped to identify an antibody targeting the 5-methylthio-ᴅ-xylofuranose AM [7,14]. The Fujifilm SILVAMP TB LAM immunoassay chip provided a threefold higher sensitivity than Alere LF-LAM. Still, current technologies lack sensitivity and are limited to patients coinfected with HIV [7,10,15]. Identification of novel anti-TB agents that specifically target AMs with high sensitivity are required. Pure AM oligosaccharides may help to identify new lead compounds for the development of diagnostics. Since the isolation of pure oligosaccharides from MTB strains in sufficient quantities is challenging, the chemical synthesis of AM is an attractive alternative. AM polysaccharides as large as 92-mers as well as many shorter AM oligomers have been prepared by solution-phase synthesis [16–20]. These multi-step syntheses are challenging and time-consuming. AGA reduces time and effort to access complex glycans [21], including linear and branched oligoarabinofuranosides α-(1,5)- and α-(1,3)- [22] as well as arabinomannose [23–24].

**Scheme 1:**
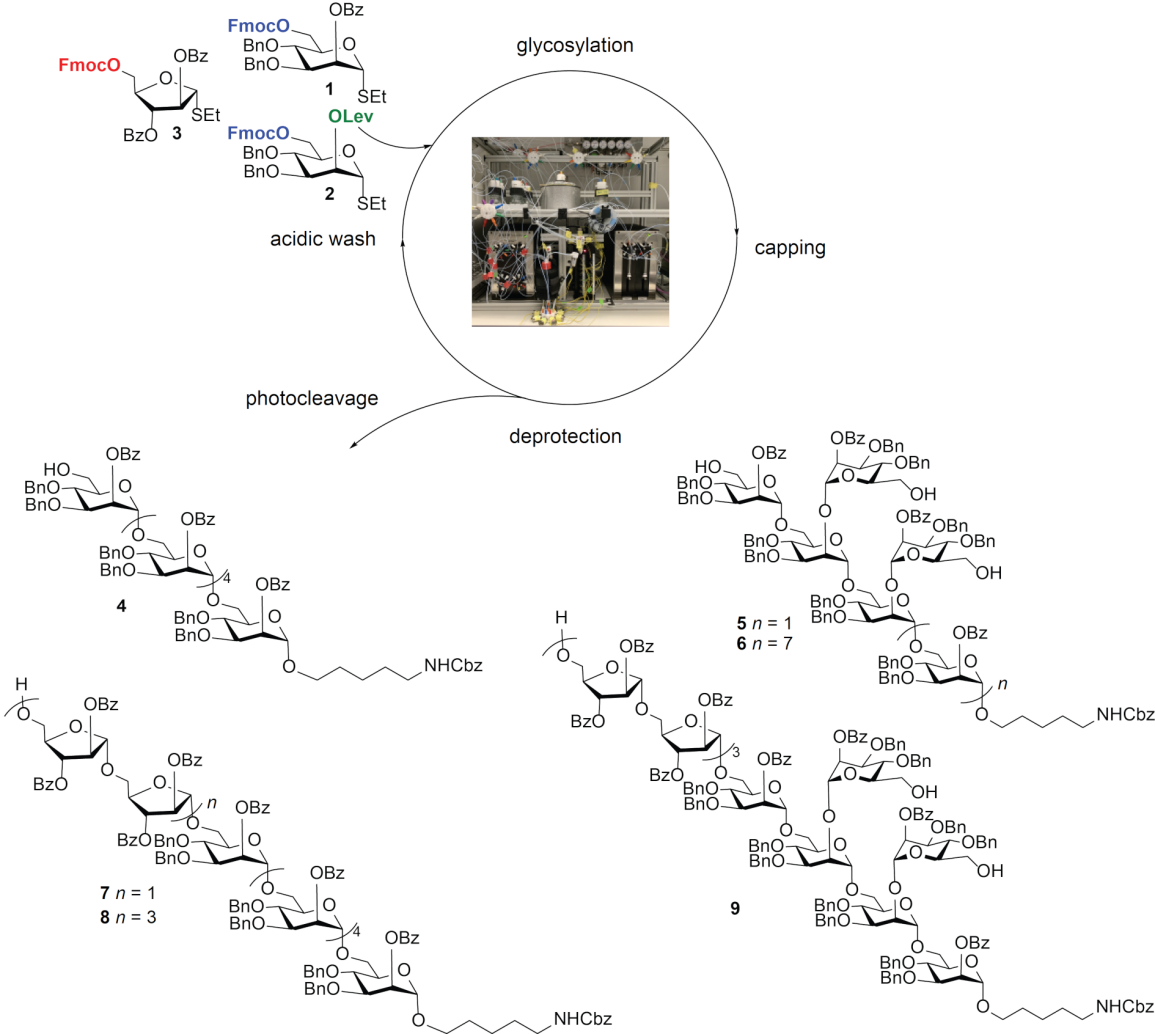
Schematic representation of AGA for oligomannosides and oligoarabinomannosides using building blocks **1**–**3**.

Herein, we describe the AGA of oligosaccharides **4**–**9** that resemble portions of the MTB AM core structure (Scheme 1). Just three building blocks, namely **1**–**3**, were required to access all six oligosaccharides, ranging from hexa- to dodecasaccharides.

## Results and Discussion

The automated syntheses of oligomannosides **4**–**6** and oligoarabinomannosides **7**–**9** were performed on a self-built automated synthesizer using a Merrifield resin functionalized with a photocleavable linker as solid support [25]. A typical AGA cycle consisted of three modules. The acidic wash module prepared the resin for glycosylation by quenching any remaining base from a previous cycle. In the glycosylation module, the thioglycoside donor was coupled to the resin upon activation with NIS and TfOH (from −40 to −20 °C). Finally, the deprotection module removed the temporary protecting group, such as fluorenylmethyloxycarbonyl (Fmoc) or levulinoyl (Lev), to reveal a free hydroxy group that allowed for further chain elongation in the next cycle. Fmoc and Lev were used as orthogonal temporary protecting groups, whereby Fmoc was cleaved by piperidine (20 vol % in DMF), while Lev was removed using hydrazine acetate. Iterative cycles continued until the desired structure was obtained. The oligosaccharide products were cleaved from the solid support using a flow UV photoreactor, followed by a two-step purification and global deprotection [25].

Linear α-(1,6)-hexamannoside **4** was synthesized using six coupling cycles and 6.5 equiv of mannose building block (BB) **1**. No deletion sequences were observed and the crude product was purified using normal-phase HPLC to obtain hexamannoside **4** in 55% yield, based on resin loading. The doubly branched hexamannoside **5** was assembled using BB **1** for α-(1,6) linkages and BB **2** for α-(1,6)/α-(1,2) branching points. First, the linear α-(1,6)-trimannose was assembled, followed by deprotection of both Lev and Fmoc to reveal three hydroxy groups. Three sequential glycosylation cycles using 6.5 equiv of BB **1** afforded compound **5** in 37% yield. Chromatographic analysis revealed compound **5** as the major product, along with pentamer and tetramer side products from incomplete glycosylation. To improve the glycosylation efficiency, a new glycosylation module employing higher incubation and reaction temperatures (from −20 °C to 0 °C) was introduced (procedure B). Capping after each glycosylation prevented the formation of undesired side products [26] and improved the isolated yield of **5** to 53%, with no detectable deletion sequences (Table 1).

**Table 1:** AGA of arabinomannosides **4**–**9**. Procedure A modules: i) acidic wash, ii) glycosylation (from −40 °C to −20 °C), and iii) Fmoc/Lev deprotection. Procedure B modules: i) acidic wash, ii) glycosylation (from −20 °C to 0 °C), iii) capping, and iv) Fmoc/Lev deprotection.

compound	procedure A yields (%)	procedure B yields (%)

**4**	55	−
**5**	37	53
**6**	6	48
**7**	9	56
**8**	7	61
**9**	3	26

The inclusion of a capping step in the AGA synthesis cycle (procedure B) was further illustrated in the synthesis of oligosaccharides **6**–**9**. AGA of the branched 12-mer mannoside **6** showed a dramatic improvement, with yields rising from 6% (procedure A) to 48% (procedure B). Syntheses of linear octamer-arabinomannoside **7** and linear 12-mer **8** were addressed using building block **1** and arabinose BB **3** for the α-(1,5)-Ara linkage. Procedure A efficiently provided the linear mannose backbone, but resulted only in partial glycosylation of arabinose BB **3**, thus giving the hexamannoside as main product as well as multiple side products missing one or more arabinoses. The desired products **7** and **8** were isolated in only 9 and 7% yield, respectively. In contrast, most deletion sequences were absent and **7** and **8** were isolated in 56 and 61% yield, respectively, when procedure B was employed.

**Figure 1:**
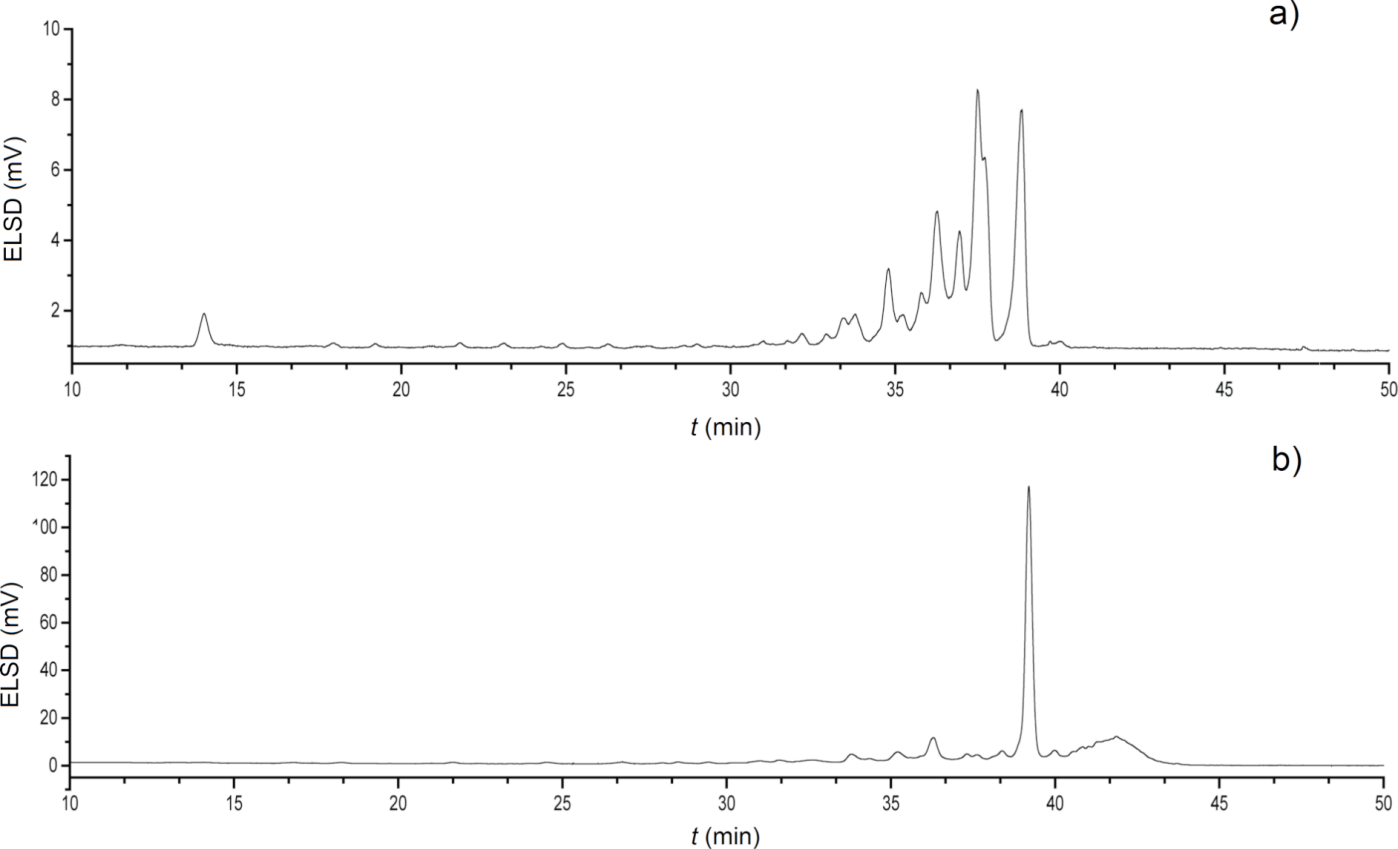
HPLC chromatograms of crude dodecamer **9**. a) Results obtained with AGA procedure A. b) Results obtained with AGA procedure B. For detailed procedures, see Tables S10 and S11 in Supporting Information File 1.

The advantage of the new procedure became more apparent for AGA of dodecamer **9**, which required all three BBs. While procedure A yielded just 3% dodecamer **9** (Figure 1a), procedure B gave **9** as the major product (26%, Figure 1b).

**Figure 2:**
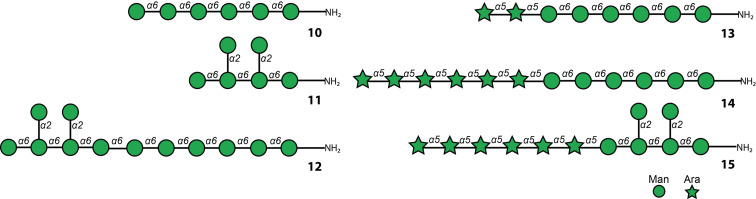
Schematic representation of LAM and AM oligomers obtained using AGA.

The global deprotection of oligosaccharides **4**–**9** was achieved by removal of the benzoate ester protecting groups under Zemplén methanolysis, followed by Pd/C-catalyzed hydrogenolysis of the carboxybenzyl group and the benzyl ethers. Mannosides **4**–**6** were deprotected and purified using reversed-phase HPLC to obtain fully deprotected mannosides **10**–**12** (Figure 2). For the arabinomannosides **7**–**9**, the acid-labile arabinose chain was cleaved during hydrogenation, giving a complex mixture of deletion sequences lacking one to six arabinose moieties. To overcome this challenge, hydrogenolysis with Pd(OH)_2_ was performed to access the fully deprotected arabinomannosides **13**–**15** (Figure 2).

## Conclusion

A collection of AM oligosaccharides containing α-(1,6)-Man, α-(1,5)-Ara, and α-(1,2)-Man was synthesized by AGA using three monosaccharide building blocks. The linear oligomannosides were readily assembled and isolated in short time and high yield under standard conditions. The introduction of a capping step after each glycosylation and optimized reaction conditions allowed for the synthesis of larger oligosaccharides. The resulting oligosaccharides will be used to study the substructure-specific antibody recognition of arabinomannans.

## Supporting Information

File 1 NMR spectra of AM and detailed information on AGA.
