# Supplementary material for: Automated glycan assembly of arabinomannan oligosaccharides from Mycobacterium tuberculosis
Source: Beilstein J Org Chem. 2019 Dec 6;15:2936–40. doi: 10.3762/bjoc.15.288 (PMC6902893; doi:10.3762/bjoc.15.288)
Supplement: File 1 — NMR spectra of AM and detailed information on AGA. [file Beilstein_J_Org_Chem-15-2936-s001.pdf]

BEILSTEIN JOURNAL OF ORGANIC CHEMISTRY

Supporting Information

for

Automated glycan assembly of arabinomannan oligosaccharides from *Mycobacterium tuberculosis*

Alonso Pardo-Vargas, Priya Bharate, Martina Delbianco and Peter H. Seeberger

Beilstein J. Org. Chem. **2019,** *15,* 2936–2940. doi:10.3762/bjoc.15.288

NMR spectra of AM and detailed information on AGA

License and Terms: This is a supporting information file under the terms of the Creative Commons Attribution License (http://creativecommons.org/licenses/by/4.0). Please note that the reuse, redistribution and reproduction in particular requires that the authors and source are credited.
The license is subject to the *Beilstein Journal of Organic Chemistry* terms and conditions: (https://www.beilstein-journals.org/bjoc)

Contents

1. General methods S2

2. Synthesis of building blocks S3

2.1. Ethyl 3,4-di-*O*-benzyl-6-*O*-fluorenylmethoxycarbonylthio-α-D-mannopyranoside (**S2**) S3

2.2. Ethyl 3,4-di-*O*-benzyl-6-*O*-fluorenylmethoxycarbonyl-2-*O*-levulinoyl-1-thio-α-D-mannopyranoside (**2**) S4

3. Pre-automation steps S6

3.1. Materials and measurements S6

3.2. Preparation of stock solutions for automated synthesis S6

4. Modules for automated synthesis S7

4.1. Post-synthesizer manipulations S9

4.2. Oligosaccharide deprotection S10

5. Experimental data S11

5.1. α(1-6) Linear hexamannoside (**4**) S11

5.2. α(1-6)/α(1-2) Branched hexamannoside (**5**) S14

5.3. α(1-6)/α(1-2) Branched dodecamannoside (**6**) S19

5.4. α(1-6)/α(1-5) Linear octaarabinomannoside (**7**) S24

5.5. α(1-6)/α(1-5) Linear dodecaarabinomannoside (**8**) S29

5.6. α(1-6)/α(1-2)/α(1-5) Branched dodecaarabinomannoside (**9**) S34

6. References S40

1. General methods

All chemicals were reagent grade and used as supplied unless otherwise noted. The automated syntheses were performed on a self-built synthesizer developed at the Max Planck Institute of Colloids and Interfaces. Analytical thin-layer chromatography (TLC) was performed on Merck silica gel 60 F254 plates (0.25 mm). Compounds were visualized by UV irradiation or dipping the plate in a *p*-anisaldehyde (PAA) solution. Flash column chromatography was carried out by using forced flow of the indicated solvent on Fluka Kieselgel 60 M (0.04–0.063 mm). Analysis and purification by normal and reversed-phase HPLC was performed by using an Agilent 1200 series. Products were lyophilized using a Christ Alpha 2-4 LD plus freeze dryer. ^{1}H, ^{13}C, and HSQC NMR chemical shifts (δ) are reported in ppm (relative to the resonance of the solvent) on a Varian 400-MR (400 MHz), Varian 600-MR (600 MHz), or Bruker Biospin AVANCE700 (700 MHz) spectrometer. Residual solvent peaks were used as internal standard ($CDCl_3$: 7.26 ppm for ^{1}H and 77.1 ppm for ^{13}C; D_2O: 4.79 ppm for ^{1}H). High-resolution mass spectra were obtained using a 6210 ESI–TOF mass spectrometer (Agilent) and MALDI–TOF autoflexTM (Bruker) instruments. IR spectra were recorded on a PerkinElmer 1600 FTIR spectrometer. Optical rotations were measured by using a PerkinElmer 241 and Unipol L1000 polarimter, with concentrations expressed in g/100 mL. The loading determination of functionalized resins was obtained using a Shimadzu UV-MINI-1240 spectrometer.

2. Synthesis of building blocks

2.1. Ethyl 3,4-di-*O*-benzyl-6-*O*-fluorenylmethoxycarbonylthio-α-D-mannopyranoside (S2)

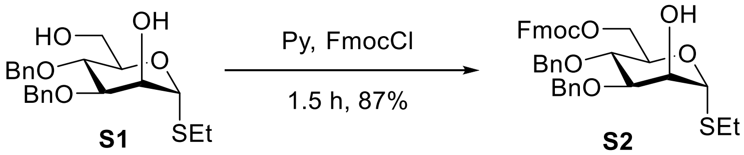

S1 **S2**

Compound **S1** [1] (1.0 g, 2.4 mmol) was dissolved in anhydrous DCM (25 mL) and pyridine (0.4 mL, 4.9 mmol), followed by the addition of FmocCl (0.7 g, 2.7 mmol). The solution was stirred at room temperature for 1.5 h, after which time the reaction mixture was dissolved in EtOAc and washed with 1 M HCl (1 × 15 mL), saturated $NaHCO_3$ (1 × 50 mL), and brine (1 × 30 mL). The organic layer was dried over Na_2SO_4, filtered, and concentrated under vacuum. The product was purified by column chromatography (10% EtOAc/hexane) to yield **S2** as a white foam (1.4 g, 87%). R_f 0.56 (EtOAc/hexane, 2:3, v/v); ^{1}H NMR (400 MHz, $CDCl_3$) δ 7.77 (d, *J* = 7.5, 1.0 Hz, 2H), 7.62 (t, *J* = 7.3, 1.0 Hz, 2H), 7.43–7.27 (m, 14H), 5.40 (s, 1H), 4.91 (dd, *J* = 11.0, 1.2 Hz, 1H), 4.70 (s, 2H), 4.63 (dd, *J* = 10.9, 1.1 Hz, 1H), 4.47–4.40 (m, 2H), 4.39–4.34 (m, 2H), 4.32–4.27 (m, 1H), 4.25 (d, *J* = 7.4 Hz, 1H), 4.12 (dt, *J* = 3.7, 1.7 Hz, 1H), 3.90–3.86 (m, 1H), 3.85–3.81 (m, 1H), 2.69–2.65 (m, 1H), 2.63–2.52 (m, 1H), 1.30 ppm (t, 3H); ^{13}C NMR (101 MHz, $CDCl_3$) δ 155.19, 143.56, 143.41, 141.37, 138.02, 137.55, 128.76, 128.61, 128.14, 128.12, 128.01, 127.97, 127.27, 125.37, 125.31, 83.40, 80.54, 75.32, 74.36, 72.22, 70.05, 69.81, 66.93, 46.80, 25.04, 14.95 ppm; HRMS (*m*/*z*): $[M + Na]^+$ calcd for $C_{37}H_{38}O_7SNa$, 649.2236; found, 649.2245.

2.2. Ethyl 3,4-di-*O*-benzyl-6-*O*-fluorenylmethoxycarbonyl-2-*O*-levulinoyl-1-thio-α-D-mannopyranoside (2)

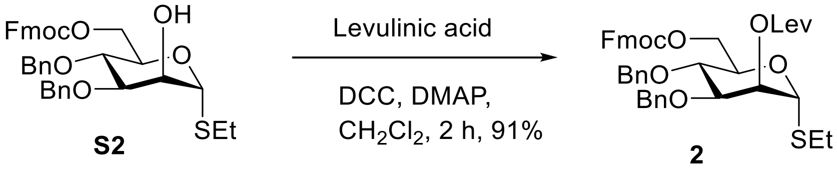

A suspension of *N*,*N*'-dicyclohexylcarbodiimide (DCC, 0.19 g, 0.94 mmol) and 4-dimethylaminopyridine (DMAP, 0.01 g, 0.09 mmol) in anhydrous DCM (3 mL) was added to a solution of **S2** (0.19 g, 0.30 mmol) and levulinic acid (0.07 ml, 0.69 mmol) in DCM (5 mL) at 0 °C. The clear solution was stirred at room temperature and monitored by TLC for 1.5 h. The mixture was filtered over celite and the filtrate concentrated under vacuum. The product was purified by column chromatography (20% ethyl acetate/hexane) to give **2** as a sticky white solid (0.2 g, 91%). R_f 0.40 (EtOAc/hexane, 1:4, v/v). IR (film) ν_{max}: 3033, 2930, 1745, 1721, 1498, 1453, 1368, 1256, 1156, 1100, 1031, 969, 851, 788, 759, 742, 700 cm^{-1}; ^1H NMR (400 MHz, $CDCl_3$) δ 7.77 (d, *J* = 7.6 Hz, 2H), 7.62 (t, *J* = 7.1 Hz, 2H), 7.40 (t, *J* = 7.5 Hz, 2H), 7.35–7.28 (m, 12H), 5.46 (s, 1H), 5.28 (s, 1H), 4.69 (d, *J* = 11.1 Hz, 1H), 4.58 (d, *J* = 10.9 Hz, 1H), 4.52 (d, *J* = 11.1 Hz, 1H), 4.43 (t, *J* = 7.0 Hz, 4H), 4.27 (q, *J* = 8.6, 7.8 Hz, 2H), 3.94 (dd, *J* = 9.3, 3.2 Hz, 1H), 3.79 (t, *J* = 9.5 Hz, 1H), 2.77–2.67 (m, 4H), 2.66–2.55 (m, 2H), 2.15 (s, 3H), 1.27 ppm (t, *J* = 7.2 Hz, 3H); ^{13}C NMR (100 MHz, $CDCl_3$) δ 206.39, 171.99, 155.17, 143.55, 143.38, 141.41, 141.39, 138.09, 137.65, 128.55, 128.33, 128.15, 128.00, 127.95, 127.27, 127.25, 125.27, 125.22, 120.17, 82.47, 78.55, 75.33, 74.32, 71.79, 70.52, 70.17, 70.00, 66.92, 46.87, 38.06, 29.88, 28.29, 25.68 ppm; HRMS (*m*/*z*): $[M + Na]^+$ calcd for $C_{42}H_{44}O_9SNa$, 747.2598; found, 747.2532.

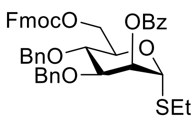

1

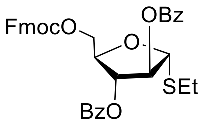

3

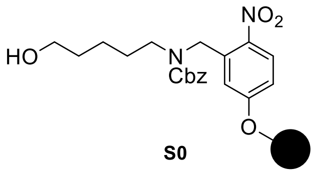

S0

Building block **1**, **3**, and photolabile resin **S0** were prepared according to reported procedures [2-5].

3. Pre-automation steps

3.1. Materials and measurements

Solvents used for dissolving all building blocks and making AGA solutions were taken from an anhydrous solvent system (jcmeyer phoenix solvent drying system). Solvents for washing were HPLC grade. The building blocks were coevaporated three times with anhydrous toluene and dried for 1 h under high vacuum prior to use. All solution bottles were freshly prepared and kept under argon during the automation process. Isolated yields of products were calculated on the basis of resin loading. Resin loading was determined following a reported protocol [1]. Briefly, functionalized resin (40 mg) was treated with one glycosylation cycle using 65 mg of BB A (excess), followed by DBU-promoted Fmoc cleavage and determination of dibenzofulvene concentration by UV absorbance. All automated syntheses were performed on a 0.0125 mmol scale. Resin was placed in the reaction vessel and was swollen in DCM for 20 min at room temperature before starting the first module. During this time, all reagent lines involved in the synthesis were washed and primed.

3.2. Preparation of stock solutions for automated synthesis

A. **Building block solution:** 0.081 mmol of building block was dissolved in 1 mL of DCM.

B. **Acidic wash solution:** 0.45 mL of TMSOTf was added to 40 mL of anhydrous DCM. The solution was kept at room temperature during the automation run.

C. **Activator solution:** Recrystallized NIS (1.35 g) was dissolved in a 2:1 mixture of anhydrous DCM and anhydrous dioxane (40.0 mL). Then, triflic acid (55 µL) was added and the resulting solution purged with Ar for 1–2 min.

D. **Fmoc deprotection solution:** A solution of 20% piperidine in DMF (v/v) was prepared.

E. **Lev deprotection solution:** Hydrazine acetate (550 mg) was dissolved in a solution of pyridine:AcOH:H_2O, 4:1:0.25, v/v/v (40 mL).

F. **Capping solution:** A solution of 10% acetic anhydride and 2% methanesulfonic acid in dry DCM (v/v) was prepared.

G. **Pre-capping solution:** A solution of 10% pyridine in DMF (v/v) was prepared.

4. Modules for automated synthesis

Module A: Preparation of resin for synthesis

All automated syntheses were performed on 0.0125 mmol scale. Resin was placed in the reaction vessel and swollen in DCM for 20 min at room temperature prior to synthesis. Before the first glycosylation, the resin was washed with the DMF, THF, and DCM (three times with 2 mL for 25 s each).

Module B: Acidic wash

The resin was swollen in 2 mL DCM and 1 mL of solution B (TMSOTf) was delivered to the reaction vessel. After 3 min at −20 °C, the solution was drained and the resin was washed with 2 mL DCM for 25 s.

Module C1: Glycosylation

The thioglycoside building block solution (6.5 equiv, 0.08 mmol in 1.0 mL DCM) was delivered to the reaction vessel and the temperature was adjusted to −20 °C. After the set temperature was reached, the reaction was started by the addition of the activator solution C. The glycosylation was performed for 5 min at −20 °C and for 20 min at 0 °C. After the glycosylation, the solution was drained and the resin was washed with DCM, DCM:dioxane (1:2, v/v, 3 mL for 20 s) and DCM (2 × 2 mL for 25 s).

Action	Cycles	Solution	Amount	*T* (°C)	Incubation time
Cooling	–	–	–	−20	–
Deliver	1	BB solution	1 mL	−20	–
Deliver	1	Activator solution	1 mL	−20	–
Reaction time (BB-dependent)	1	–	–	−20<br>to 0	5 min<br>20 min
Wash	1	DCM	2 mL	0	5 s
Wash	1	DCM : Dioxane (1:2, v/v)	2 mL	0	20 s
Heating	–	–	–	25	–
Wash	2	DCM	2 mL	>0	25 s

Module C2: Glycosylation

The thioglycoside building block solution (6.5 equiv, 0.08 mmol in 1.0 mL DCM) was delivered to the reaction vessel and the temperature was adjusted to −40 °C. After the set temperature was reached, the reaction was started by the addition of the activator solution C. The glycosylation was performed for 5 min at −40 °C and for 30 min at −20 °C. After the glycosylation, the solution was drained and the resin was washed with DCM, DCM:dioxane (1:2, v/v, 3 mL for 20 s) and DCM (two times, each with 2 mL for 25 s).

Module D: Fmoc deprotection

The resin was washed with DMF (three times with 2 mL for 25 s) and the temperature of the reaction vessel was adjusted to 25 °C. Fmoc deprotection solution (2 mL) was delivered into the reaction vessel. After 5 min, the reaction solution was drained and the resin washed with DMF (three times with 3 mL for 25 s) and DCM (five times each with 2 mL for 25 s).

Module E: Lev deprotection

The resin was washed with DMF (3 × 30 min) and 1.3 mL DCM was added to the reaction vessel. Solution E (0.8 mL) was added to the reaction vessel, and the temperature was adjusted to 25 °C. After 30 min, the reaction solution was drained and the whole cycle was repeated two more times. After Lev deprotection was complete, the resin was washed with DMF, THF, and DCM.

Module F: Capping

The resin was washed with DMF (two times with 2 mL for 25 s) and the temperature was adjusted to 25 °C. 2 mL of solution G was delivered into the reaction vessel. After 1 min, the reaction solution was drained and the resin was washed with DCM (three times with 3 mL for 25 s). 4 mL of capping solution F was delivered into the reaction vessel. After 20 min, the reaction solution was drained and the resin washed with DCM (three times with 3 mL for 25 s) [6].

4.1. Post-synthesizer manipulations

Cleavage from the solid support: The oligosaccharides were cleaved from the solid support using the Vapourtec E-Series UV-150 photoreactor as reported previously [7].

Purification: The crude products were analyzed and purified using analytical and preparative HPLC (Agilent 1200 Series spectrometer).

- **Method A:** (YMC-Diol-300 column, 150 × 4.6 mm) flow rate of 1.0 mL/min with hexane/20% EtOAc as eluent [isocratic 20% EtOAc (5 min), linear gradient to 55% EtOAc (45 min), linear gradient to 100% EtOAc (5 min)].
- **Method B:** (YMC-Diol-300 column, 150 × 20 mm) flow rate of 15 mL/min with hexane/20% EtOAc as eluents [isocratic 20% EtOAc (5 min), linear gradient to 55% EtOAc (35 min), linear gradient to 100% EtOAc (5 min)].
- **Method C:** (Hypercarb column, 150 × 4.6 mm) flow rate of 0.7 mL/min with H_2O (0.1% formic acid) as eluents [isocratic (5 min), linear gradient to 30% acetonitrile (30 min), linear gradient to 100% acetonitrile (5 min)].
- **Method D:** (Hypercarb column, 150 × 10 mm) flow rate of 1.3 mL/min with H_2O (0.1% formic acid) as eluents [isocratic (5 min), linear gradient to 30% acetonitrile (30 min), linear gradient to 100% acetonitrile (5 min)].
- **Method E:** (Synergi Hydro RP18 column, 250 × 4.6 mm) flow rate of 1.0 mL/min with H_2O (0.1% formic acid) as eluents [isocratic (5 min), linear gradient to 30% acetonitrile (30 min), linear gradient to 100% acetonitrile (5 min)].
- **Method F:** (Synergi Hydro RP18 column, 250 × 10 mm) flow rate of 4.0 mL/min with H_2O (0.1% formic acid) as eluents [isocratic (5 min), linear gradient to 30% acetonitrile (30 min), linear gradient to 100% acetonitrile (5 min)].

4.2. Oligosaccharide deprotection

Module G: Methanolysis

The protected oligosaccharide was dissolved in MeOH:DCM (1.5 mL, 1:1, v/v). NaOMe in MeOH (0.1 mL of a 0.5 M solution) was added to the solution and stirred at room temperature. After 12 h, the solution was neutralized with Amberlite IR-120 (H^+ form) resin, filtered and concentrated in vacuo*.* The crude compound was used for hydrogenolysis without further purification.

Module H: Hydrogenolysis with Pd/C

The crude compound obtained from module G was dissolved in 2 mL of DCM:*t*-BuOH:H_2O (2:1:1, v/v/v), and Pd/C (10%) was added. The reaction was stirred in a H_2 bomb with 60 psi pressure for 16 h. The reaction was filtered, washed with DCM, *t*-BuOH, and H_2O. The filtrates were concentrated in vacuo.

Module I: Hydrogenolysis with $Pd(OH)_2/C$

The crude compound obtained from module G was dissolved in 2 mL of EtOAc:*t*-BuOH:H_2O, 2:1:1, v/v/v and $Pd(OH)_2/C$ was added. The reaction was stirred in a H_2 bomb with 60 psi pressure for 16 h. The reaction was filtered, washed with EtOAc, t-BuOH, and H_2O. The filtrates were concentrated in vacuo.

5. Experimental data

5.1. α(1-6) Linear hexamannoside (**4**)

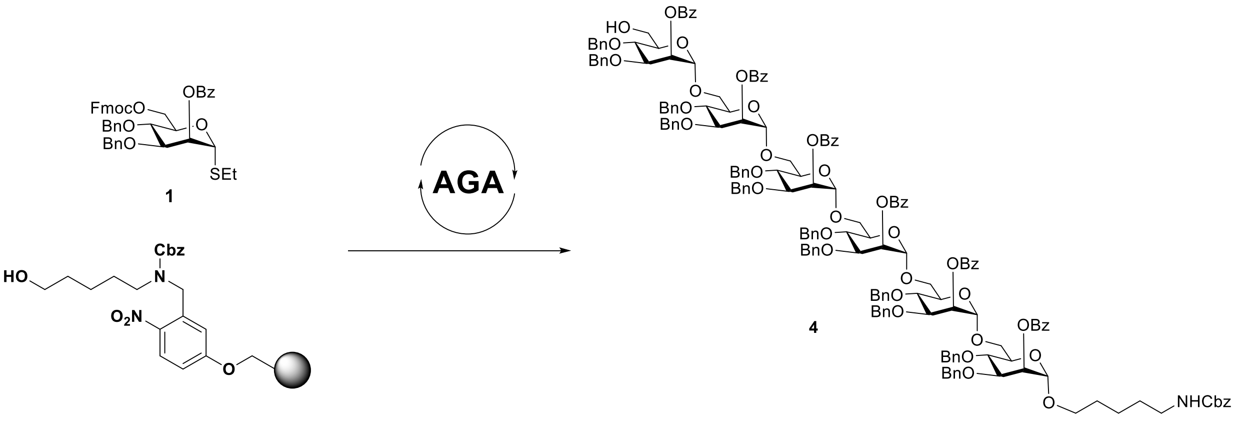

Table S1: Procedure A

	Module	Conditions
	A: Resin Preparation for Synthesis	
6	B: Acidic Wash with TMSOTf Solution	
	C2: Thioglycoside Glycosylation	BB **1** 6.5 equiv, −40° for 5 min, −20° for 30 min
	D: Fmoc Deprotection	

Cleavage from the solid support, as described in Post-synthesizer manipulations, followed by purification using preparative HPLC (Method B) afforded the fully protected hexasaccharide **4** (20 mg, 55%).

Crude NP-HPLC of hexasaccharide **4** (ELSD trace, Method A, t_R = 25.3 min).

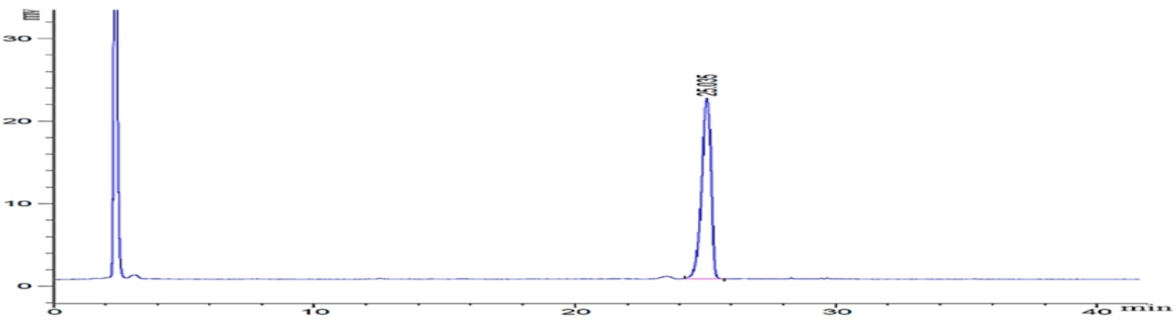

^{1}H NMR (400 MHz, $CDCl_3$) δ 8.24–8.09 (m, 12H), 7.64 (s, 1H), 7.56–7.46 (m, 17H), 7.38–7.30 (m, 12H), 7.29–7.10 (m, 56H), 5.85 (d, *J* = 9.0 Hz, 4H), 5.80 (s, 1H), 5.66

(s, 1H), 5.14 (s, 1H), 5.12–5.03 (m, 6H), 4.95–4.73 (m, 12H), 4.61 (dd, *J* = 15.6, 11.1 Hz, 2H), 4.52–4.41 (m, 7H), 4.37 (d, *J* = 12.4 Hz, 2H), 4.13–3.86 (m, 13H), 3.84–3.59 (m, 11H), 3.58–3.52 (m, 2H), 3.46 (dd, *J* = 16.9, 9.7 Hz, 4H), 3.22–3.16 (m, 2H), 1.64–1.57 (m, 2H), 1.55–1.47 (m, 2H), 1.41–1.34 ppm (m, 2H); ^{13}C NMR (100 MHz, $CDCl_3$) δ 165.97, 165.73, 165.65, 165.54, 156.51, 138.63, 138.58, 138.41, 138.37, 138.04, 137.74, 137.67, 137.65, 137.62, 136.78, 133.45, 133.41, 130.08, 130.03, 129.99, 128.77, 128.70, 128.62, 128.47, 128.43, 128.34, 128.29, 128.26, 128.18, 128.14, 127.82, 127.78, 127.75, 127.53, 127.48, 127.44, 127.34, 127.24, 127.22, 98.58, 98.51, 98.25, 97.99, 78.72, 78.42, 78.37, 78.34, 78.28, 77.79, 76.84, 75.31, 75.22, 75.16, 75.12, 74.30, 74.01, 73.91, 73.85, 73.79, 72.20, 71.76, 71.53, 71.43, 71.30, 71.09, 71.01, 70.84, 69.16, 68.65, 68.52, 68.46, 67.89, 66.68, 66.20, 65.91, 65.82, 65.54, 61.93, 41.07, 29.90, 29.16, 23.55 ppm; HRMS (*m*/*z*): $[M + Na]^+$ calcd for $C_{175}H_{175}NO_{39}Na$, 2937.163; found, 2937.160. Proton and carbon NMR signals are in agreement with previous reports by Delbianco and co-workers [5].

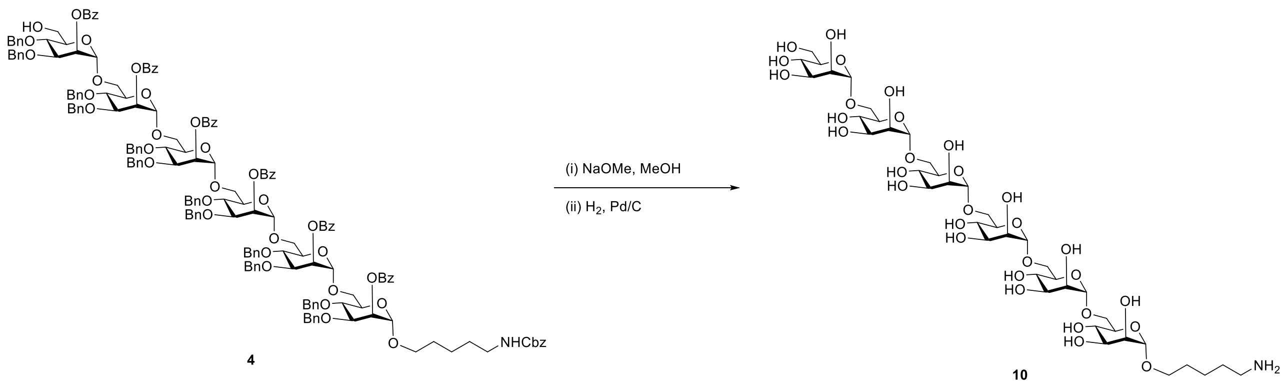

Deprotection of **4** (as described in Modules G and H), followed by purification using preparative HPLC (Method E, t_R = 22.2 min) afforded compound **10** (3.9 mg, 52%).

RP-HPLC of purified hexasaccharide **10** (ELSD trace, Method C, t_R = 22.2 min).

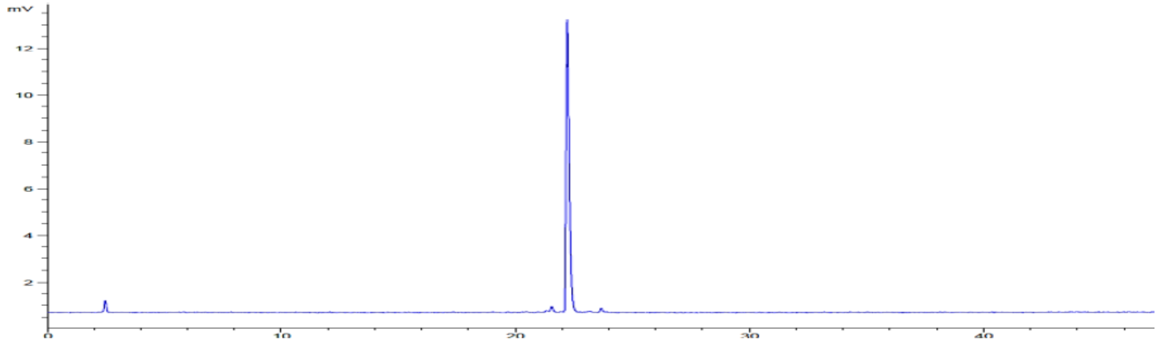

Analytical data for **10**. ^{1}H NMR (400 MHz, D_2O) δ 4.89–4.86 (m, 5H), 4.83 (s, 1H), 3.98–3.95 (m, 5H), 3.94–3.88 (m, 7H), 3.85 (s, 1H), 3.84–3.81 (m, 5H), 3.80–3.77 (m, 5H), 3.76–3.71 (m, 8H), 3.70 (d, J = 2.3 Hz, 2H), 3.69–3.60 (m, 4H), 3.57–3.51 (m, 1H), 2.98 (t, J = 7.6 Hz, 2H), 1.71–1.60 (m, 4H), 1.50–1.37 ppm (m, 2H); ^{13}C NMR (100 MHz, D_2O) δ 99.89, 99.42, 99.30, 72.72, 70.92, 70.84, 70.80, 70.78, 70.72, 70.69, 70.55, 70.07, 69.98, 69.94, 67.63, 66.75, 66.61, 66.57, 65.61, 65.58, 65.53, 60.94, 39.38, 28.04, 26.57, 22.54 ppm; HRMS (*m*/*z*): $[M + H]^+$ calcd for $C_{41}H_{73}NO_{31}$, 1076.423; found, 1076.424. Proton and carbon NMR signals are in accordance with previously reported data by Delbianco and co-workers [5].

5.2. α(1-6) α(1-2) branched hexamannoside (**5**)

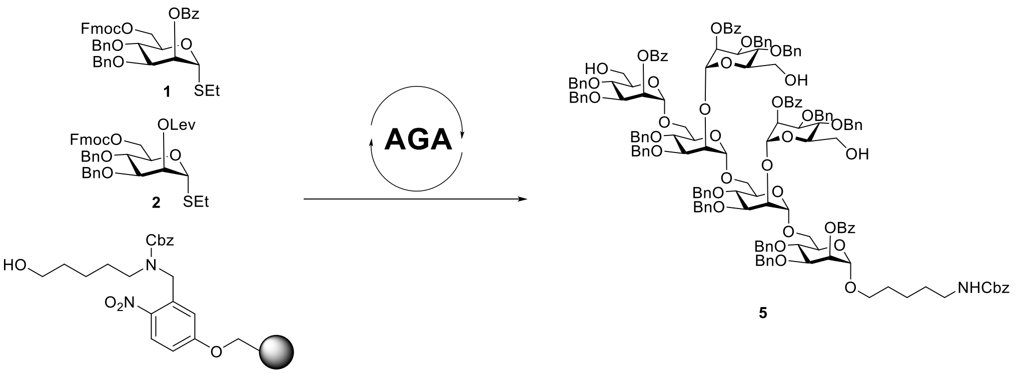

Table S2: Procedure A

	Module	Conditions
	A: Resin Preparation for Synthesis	
	B: Acidic Wash with TMSOTf Solution<br>C2: Thioglycoside Glycosylation<br>D: Fmoc Deprotection	BB **1** 6.5 equiv, −40° for 5 min, −20° for 30 min
**2**	B: Acidic Wash with TMSOTf Solution<br>C2: Thioglycoside Glycosylation<br>D: Fmoc Deprotection	BB **1** 6.5 equiv, −40° for 5 min, −20° for 30 min
	E: Lev Deprotection<br>B: Acidic Wash with TMSOTf Solution<br>3 × C2: Thioglycoside Glycosylation<br>D: Fmoc Deprotection	BB **1** 13 equiv, −40° for 5 min, −20° for 30 min

Table S3: Procedure B

	Module	Conditions
	A: Resin Preparation for Synthesis	
	B: Acidic Wash with TMSOTf Solution<br>C1: Thioglycoside Glycosylation<br>F: Capping<br>D: Fmoc Deprotection	BB **1** 6.5 equiv, −20° for 5 min, 0° for 20 min
**2**	B: Acidic Wash with TMSOTf Solution<br>C1: Thioglycoside Glycosylation<br>F: Capping<br>D: Fmoc Deprotection	BB **2** 6.5 equiv, −20° for 5 min, 0° for 20 min
	E: Lev Deprotection<br>B: Acidic Wash with TMSOTf Solution<br>3 × C1: Thioglycoside Glycosylation<br>F: Capping<br>D: Fmoc Deprotection	BB **1** 6.5 equiv, −20° for 5 min, 0° for 20 min

Cleavage from the solid support, as described in Post-synthesizer manipulations, followed by purification using a preparative HPLC (Method B), to provide the fully protected branched hexasaccharide **5**. For procedure A: 12.5 mg, 4.62 µmol, 37%, based on resin loading. For procedure B: 18.0 mg, 6.65 µmol, 53%, based on resin loading.

Crude NP-HPLC of hexasaccharide **5**. A) Procedure A. B) Procedure B (ELSD trace, Method A, t_R = 28.5 min).

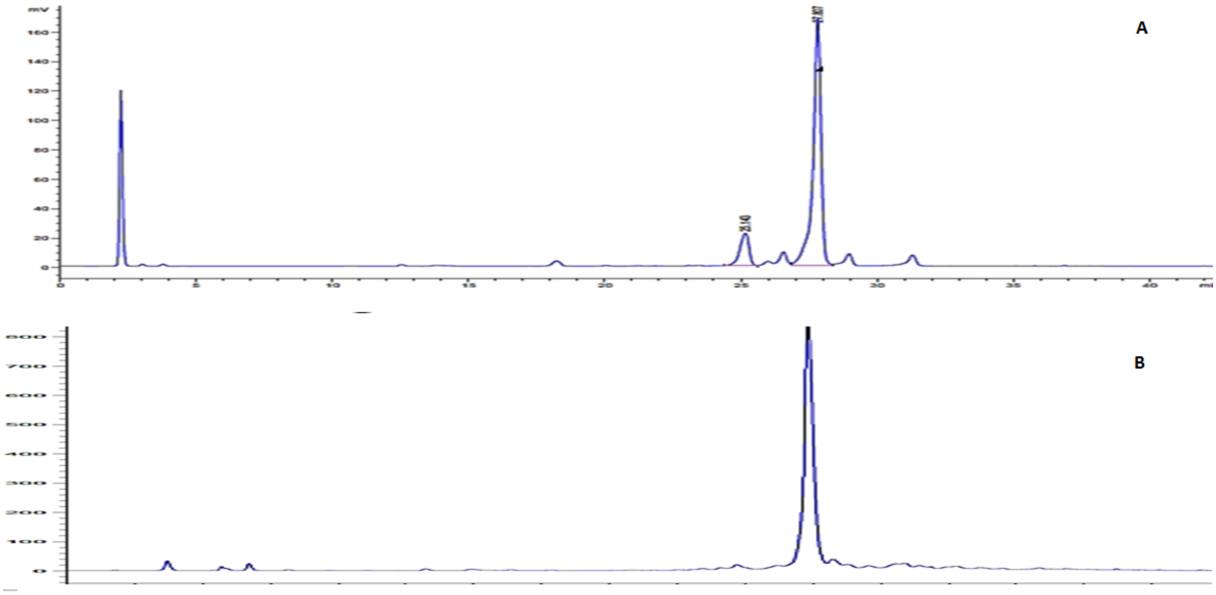

^{1}H NMR (400 MHz, $CDCl_3$) δ 8.08–7.86 (m, 9H), 7.55–7.45 (m, 4H), 7.41–7.30 (m, 8H), 7.27–6.92 (m, 65H), 5.83–5.79 (m, 1H), 5.61 (t, *J* = 2.4 Hz, 1H), 5.54 (t, *J* = 2.3 Hz, 1H), 5.45–5.40 (m, 1H), 5.23 (s, 1H), 5.11 (s, 1H), 5.03–4.97 (m, 3H), 4.85–4.81 (m, 3H), 4.79–4.67 (m, 7H), 4.62–4.26 (m, 14H), 4.17–4.09 (m, 2H), 4.02–3.90 (m, 6H), 3.87–3.75 (m, 9H), 3.72–3.48 (m, 14H), 3.44–3.27 (m, 5H), 3.11–3.02 (m, 2H), 1.48–1.38 (m, 4H), 1.26 ppm (d, *J* = 9.1 Hz, 2H); ^{13}C NMR (100 MHz, $CDCl_3$) δ 165.99, 165.52, 165.43, 165.38, 156.51, 138.66, 138.53, 138.49, 138.29, 138.21, 138.10, 138.04, 137.90, 137.81, 137.66, 137.14, 136.79, 136.76, 133.52, 133.43, 133.27, 133.22, 130.27, 130.05, 129.99, 129.90, 129.78, 129.34, 128.63, 128.55, 128.43, 128.35, 128.28, 128.16, 128.14, 128.02, 127.97, 127.88, 127.81, 127.77, 127.71, 127.56, 127.47, 127.34, 100.24, 99.88, 99.28, 98.87, 97.64, 97.55, 79.24, 79.11, 78.97, 78.61, 78.50, 75.33, 75.21, 75.12, 74.93, 74.59, 74.42, 74.10, 73.99, 73.40, 72.78, 72.49, 71.91, 71.81, 71.75, 71.39, 71.24, 71.06, 70.49, 69.62, 69.57, 69.23,

68.81, 67.84, 66.69, 66.02, 65.87, 62.59, 62.27, 61.93, 41.05, 29.86, 29.14, 23.54 ppm; HRMS (*m*/*z*): $[M + H]^+$ calcd. for $C_{161}H_{168}NO_{37}$, 2708.136; found, 2708.198.

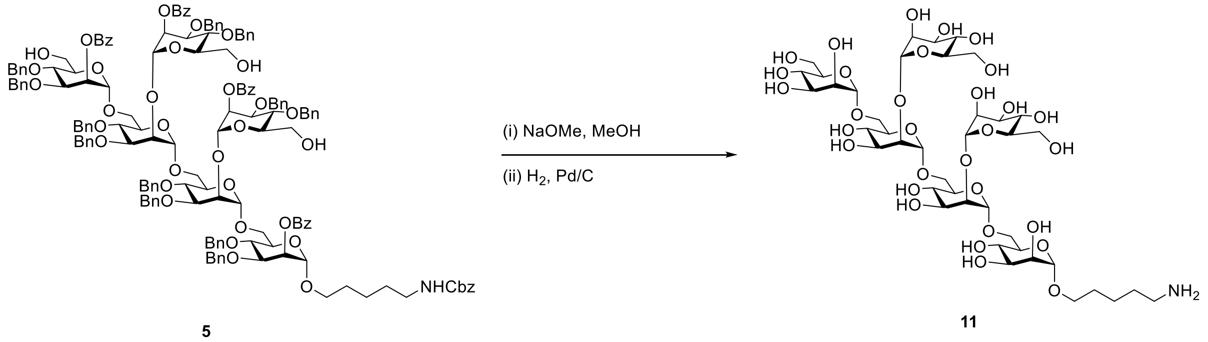

5 **11**

Deprotection of **5**, as described in Modules G and H, followed by purification using preparative HPLC (Method E) afforded compound **11** (3.1 mg, 2.88 μmol, 62% over two steps). ^{1}H NMR (600 MHz, D_2O) δ 5.15 (s, 1H), 5.14 (s, 1H), 5.06 (s, 1H), 5.05 (s, 1H), 4.95 (s, 1H), 4.87 (s, 1H), 4.12–3.54 (m, 38H), 3.02 (t, *J* = 7.7 Hz, 2H), 1.74–1.64 (m, 4H), 1.52–1.41 ppm (m, 2H); ^{13}C NMR (150 MHz, D_2O) δ 102.23 , 99., 99., 98., 78.72, 73.24, 73.17, 72.73, 71.17, 71.08, 70.83, 70.57, 70.38, 69.93, 67.55, 66.64, 66.61, 66.59, 66.50, 66.42, 65.78, 65.58, 65.11, 60.99, 60.96, 60.86, 39.29, 27.95, 26.49, 22.42 ppm; HRMS (*m*/*z*): $[M + H]^+$ calcd for $C_{41}H_{73}NO_{31}$, 1076.423; found, 1076.425.

RP-HPLC of purified hexasaccharide **11** (ELSD trace, Method C, t_R = 21.3 min).

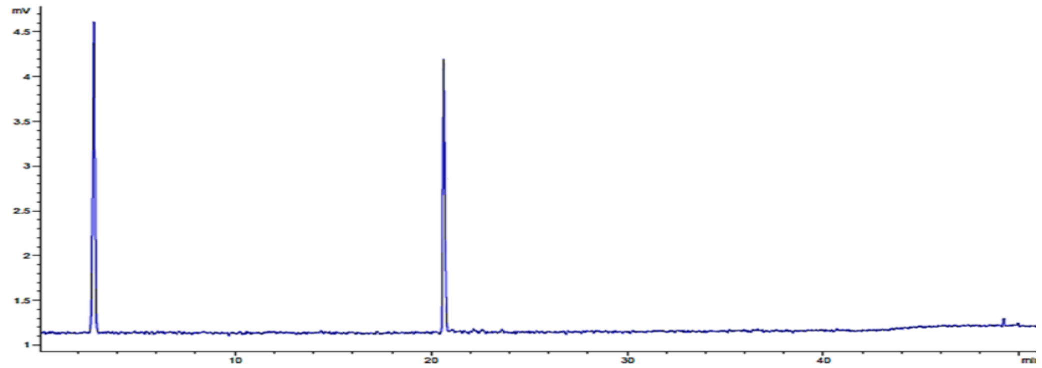

^{1}H NMR of 11 (400 MHz, D_2O)

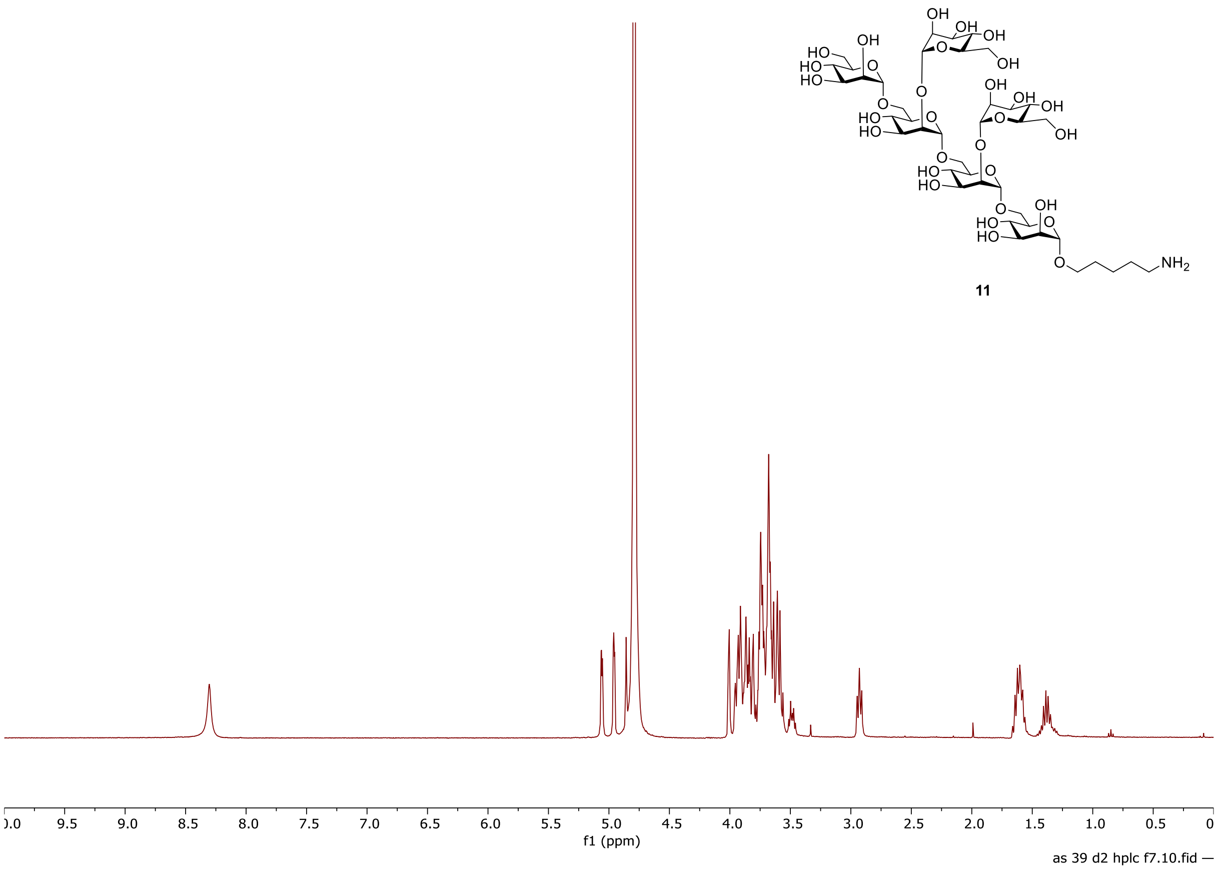

^{13}C NMR of 11 (100 MHz, D_2O)

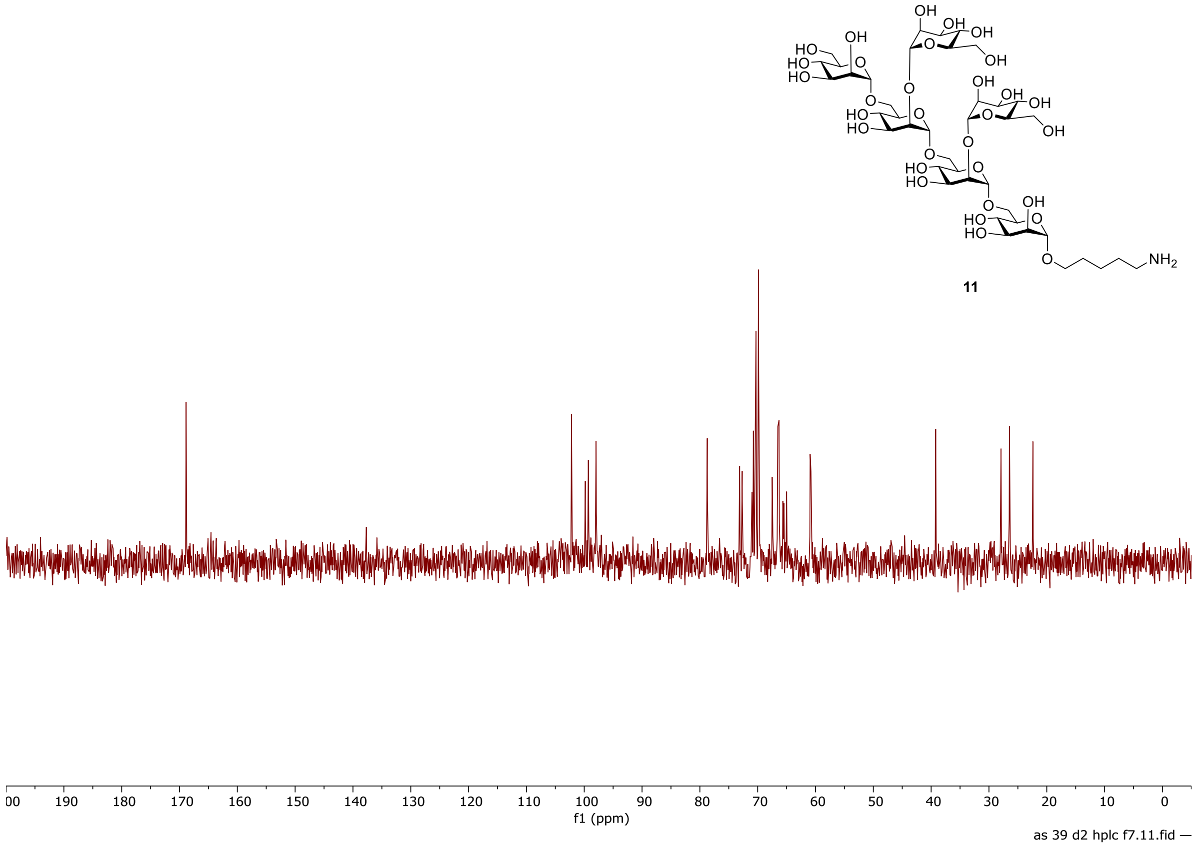

HSQC NMR of 11 (D_2O)

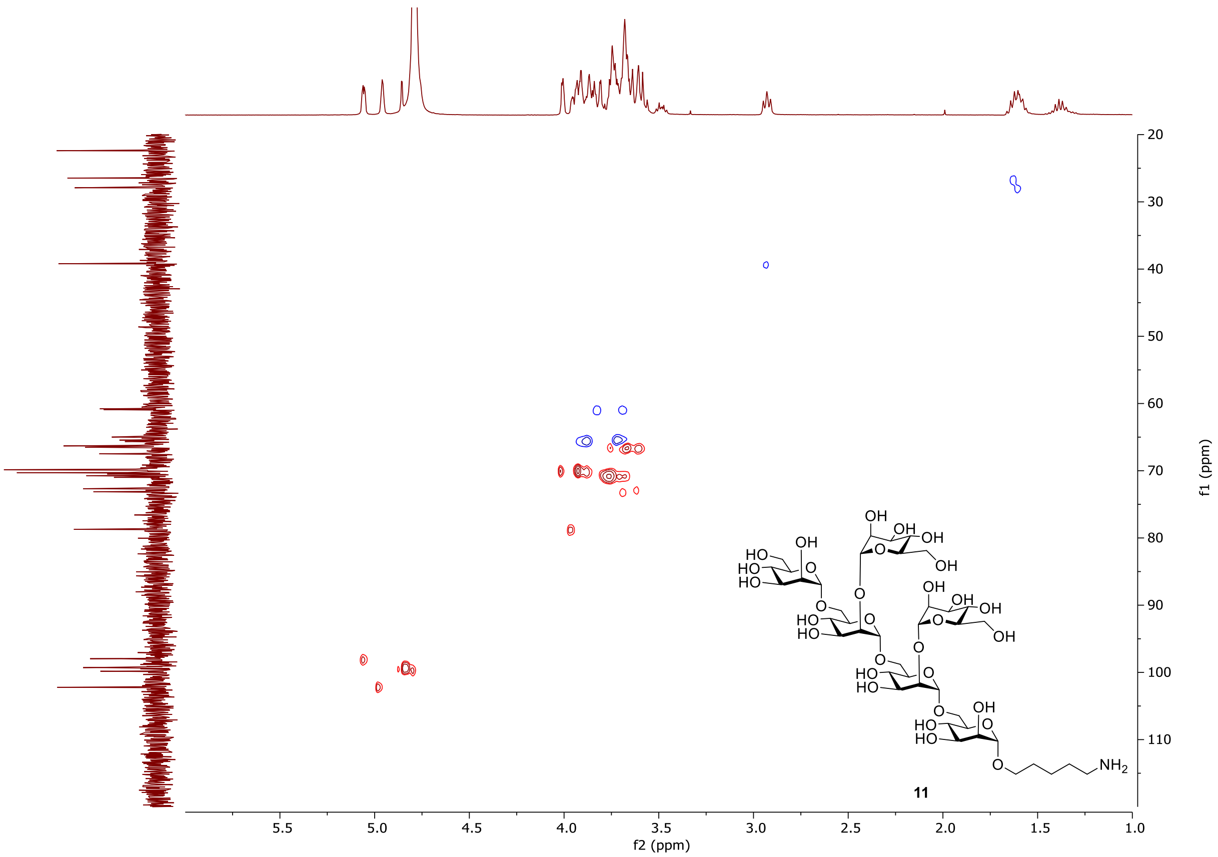

5.3. α(1-6) α(1-2) branched dodecamannoside (**6**)

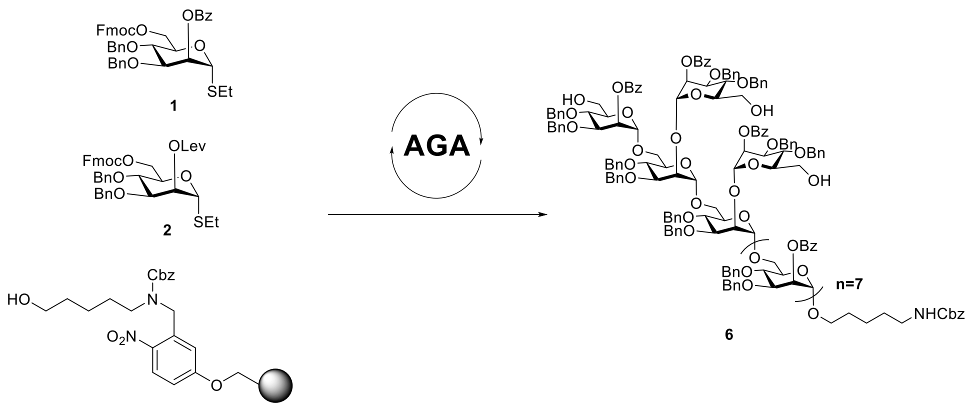

Table S4: Procedure A

	Module	Conditions
	A: Resin Preparation for Synthesis	
**7**	B: Acidic Wash with TMSOTf Solution<br>C2: Thioglycoside Glycosylation<br>D: Fmoc Deprotection	BB **1** 6.5 equiv, −40° for 5 min, −20° for 30 min
**2**	B: Acidic Wash with TMSOTf Solution<br>C2: Thioglycoside Glycosylation<br>D: Fmoc Deprotection	BB **2** 6.5 equiv, −40° for 5 min, −20° for 30 min
	E: Lev Deprotection<br>B: Acidic Wash with TMSOTf Solution<br>3 × C2: Thioglycoside Glycosylation<br>D: Fmoc Deprotection	BB **1** 13 equiv, −40° for 5 min, −20° for 30 min

Table S5: Procedure B:

	Module	Conditions
	A: Resin Preparation for Synthesis	
**7**	B: Acidic Wash with TMSOTf Solution<br>C1: Thioglycoside Glycosylation<br>F: Capping<br>D: Fmoc Deprotection	BB **1** 6.5 equiv, −20° for 5 min, 0° for 20 min
**2**	B: Acidic Wash with TMSOTf Solution<br>C1: Thioglycoside Glycosylation<br>F: Capping<br>D: Fmoc Deprotection	BB **2** 6.5 equiv, −20° for 5 min, 0° for 20 min
	E: Lev Deprotection<br>B: Acidic Wash with TMSOTf Solution<br>3 × C1: Thioglycoside Glycosylation<br>F: Capping<br>D: Fmoc Deprotection	BB **1** 6.5 equiv, −20° for 5 min, 0° for 20 min

Cleavage from the solid support, as described in Post-synthesizer manipulations, followed by purification using a preparative HPLC (Method B) to provide the fully protected branched dodecasaccharide **6**. For procedure A, 2.2 mg, 0.75 µmol, 6%, based on resin loading. For procedure B, 30 mg, 6.05 µmol, 48%, based on resin loading.

Crude NP-HPLC of dodecasaccharide **6**. A) Procedure A. B) Procedure B (ELSD trace, Method A, t_R = 31.3 min)

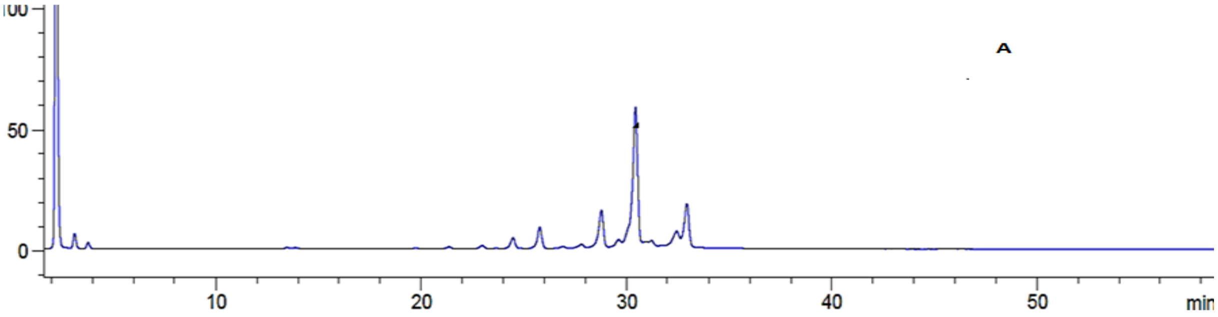

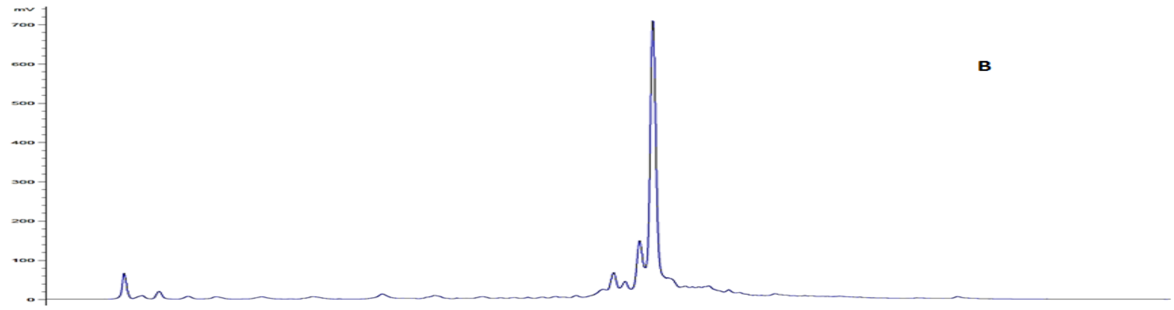

^{1}H NMR (600 MHz, $CDCl_3$) δ 8.18–8.12 (m, 8H), 8.11–8.08 (m, 2H), 8.06–8.00 (m, 6H), 7.96 (d, J = 7.7 Hz, 2H), 7.57 (t, J = 7.5 Hz, 1H), 7.54–7.34 (m, 25H), 7.33–7.00 (m, 116H), 5.88 (s, 1H), 5.82–5.78 (m, 4H), 5.71 (s, 1H), 5.67 (s, 1H), 5.64–5.60 (m, 2H), 5.30 (s, 1H), 5.12 (s, 1H), 5.09–5.01 (m, 8H), 4.92 (s, 1H), 4.90–4.74 (m, 20H), 4.67 (dd, J = 10.9, 6.7 Hz, 2H), 4.63–4.29 (m, 25H), 4.26 (s, 1H), 4.22 (dd, J = 9.0, 3.1 Hz, 1H), 4.12–3.98 (m, 11H), 3.95–3.30 (m, 45H), 3.15 (s, 2H), 1.50–1.45 (m, 4H), 1.35–1.32 ppm (m, 2H); ^{13}C NMR (152 MHz, $CDCl_3$) δ 165.99, 165.75, 165.73, 165.67, 165.53, 165.44, 165.39, 165.31, 156.52, 138.75, 138.61, 138.57, 138.49, 138.39, 138.31, 138.25, 138.08, 138.00, 137.91, 137.79, 137.73, 137.69, 137.63, 137.57, 136.82, 133.44, 133.21, 130.14, 130.06, 130.00, 129.90, 129.81, 128.78, 128.71, 128.62, 128.61, 128.47, 128.40, 128.31, 128.17, 128.11, 127.88, 127.81, 127.77, 127.56, 127.50, 127.38, 127.26, 127.11, 100.32, 99.57, 99.33, 99.04, 98.70, 98.65, 98.55, 98.43, 98.27, 98.01, 97.70, 78.73, 78.44, 78.37, 78.31, 78.24, 75.32, 75.19, 75.12, 74.34, 74.05, 73.95, 73.90, 73.59, 71.96, 71.86, 71.78, 71.68, 71.55, 71.47,

71.44, 71.38, 71.24, 71.20, 71.10, 71.01, 70.96, 70.87, 69.21, 68.89, 68.81, 68.71, 68.59, 68.54, 67.91, 66.69, 66.25, 65.95, 65.88, 41.10, 29.86, 29.18, 23.58 ppm. HRMS (*m*/*z*): $[2M + Na]^+$ calcd $C_{323}H_{323}NO_{73}Na$, 2716.074; found, 2716.056.

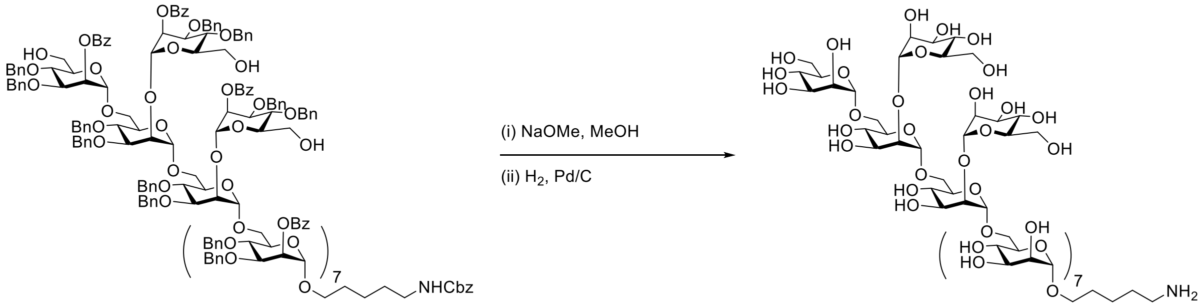

6 **12**

Deprotection of **6** as described in Modules G and H, followed by purification using preparative HPLC (Method E) afforded compound **12** (2.3 mg, 1.21 µmol, 20% over two steps). ^1H NMR (700 MHz, D_2O) δ 5.16 (s, 1H), 5.15 (s, 1H), 5.08–5.06 (m, 2H), 4.96 (s, 1H), 4.92 (d, *J* = 6.1 Hz, 5H), 4.89 (s, 1H), 4.12–4.10 (m, 2H), 4.06 (s, 2H), 4.02 (d, *J* = 4.6 Hz, 8H), 4.00–3.95 (m, 8H), 3.94–3.91 (m, 3H), 3.90–3.82 (m, 19H), 3.81–3.74 (m, 20H), 3.73–3.66 (m, 5H), 3.60 (d, *J* = 10.1, 6.1 Hz, 1H), 3.02 (t, *J* = 7.6 Hz, 2H), 1.75–1.67 (m, 4H), 1.53–1.42 ppm (m, 2H); ^{13}C NMR (175 MHz, D_2O) δ 102.28, 102.20, 99.87, 99.42, 99.40, 99.33, 99.28, 98.16, 98.07, 80.47, 78.79, 78.71, 76.13, 73.46, 73.26, 73.21, 72.78, 72.36, 71.20, 71.11, 70.99, 70.90, 70.88, 70.81, 70.77, 70.66, 70.61, 70.59, 70.42, 70.19, 70.04, 69.99, 69.96, 69.79, 69.03, 67.61, 67.24, 66.81, 66.69, 66.63, 66.56, 66.54, 66.47, 65.63, 65.54, 65.48, 65.16, 61.18, 61.05, 61.00, 60.91, 39.37, 28.02, 26.65, 24.46, 23.23, 22.53 ppm. HRMS (*m*/*z*): $[M + H]^+$ calcd for $C_{71}H_{123}NO_{56}$, 1886.688; found, 1886.677.

RP-HPLC of purified dodecasaccharide **12** (ELSD trace, Method C, t_R = 24.2 min)

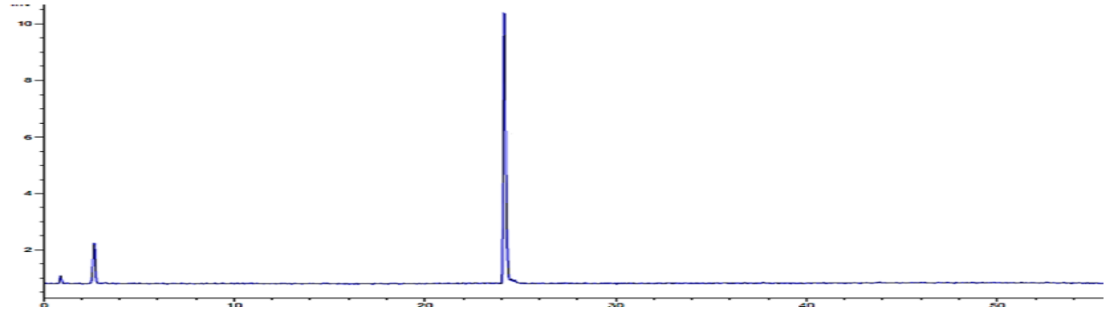

^{1}H NMR of 12 (400 MHz, D_2O)

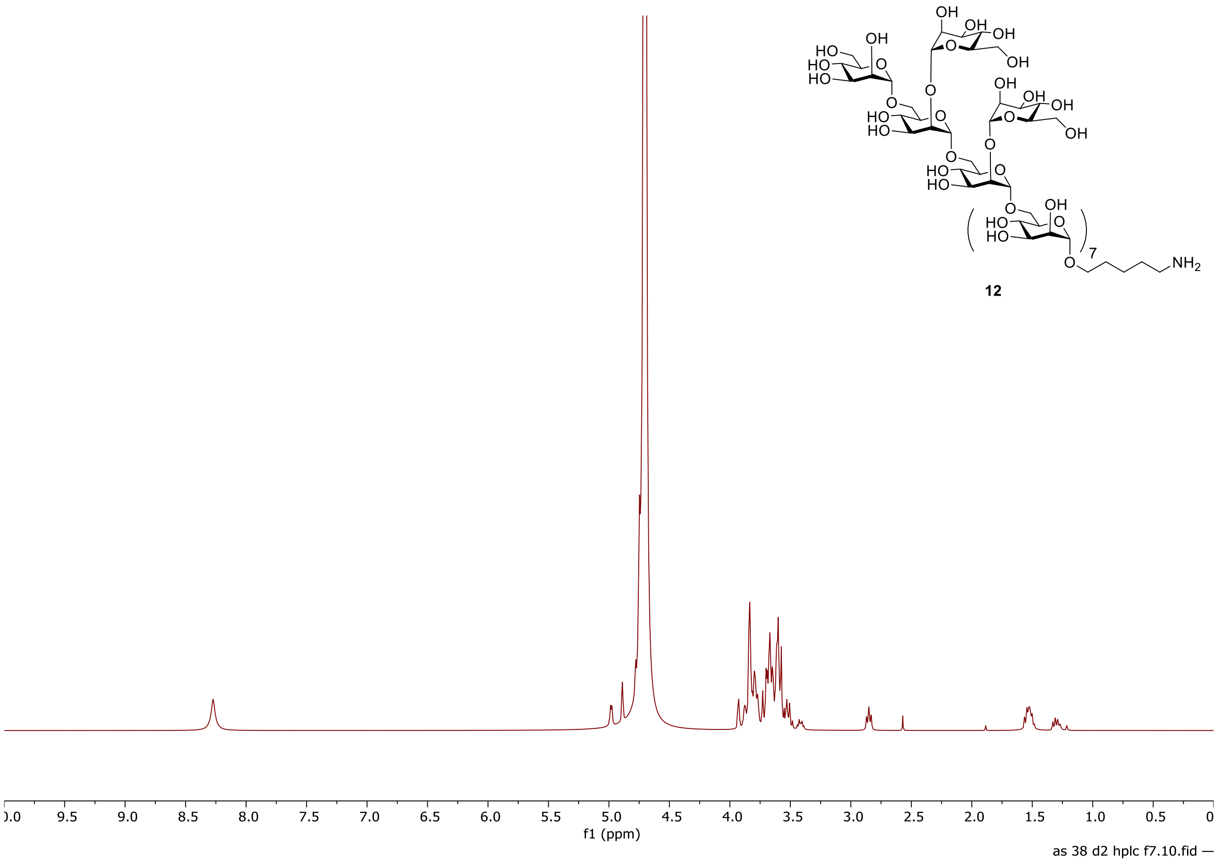

^{13}C NMR of 12 (175 MHz, D_2O)

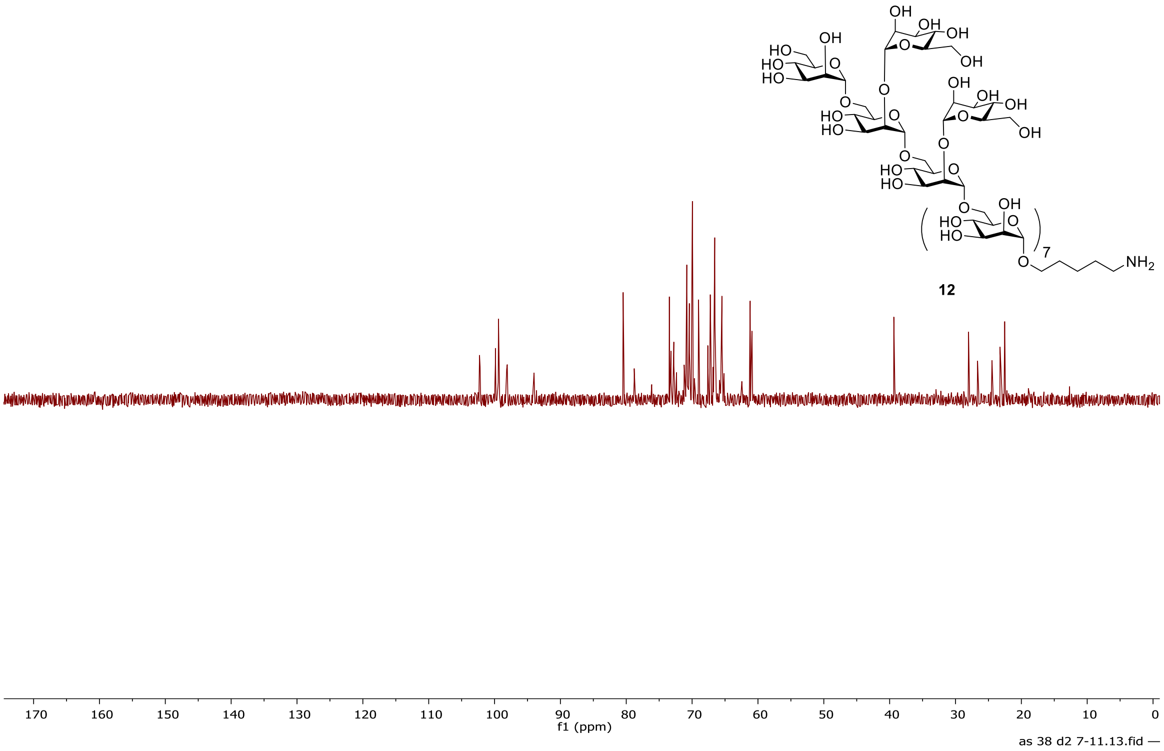

HSQC NMR of 12 (D_2O)

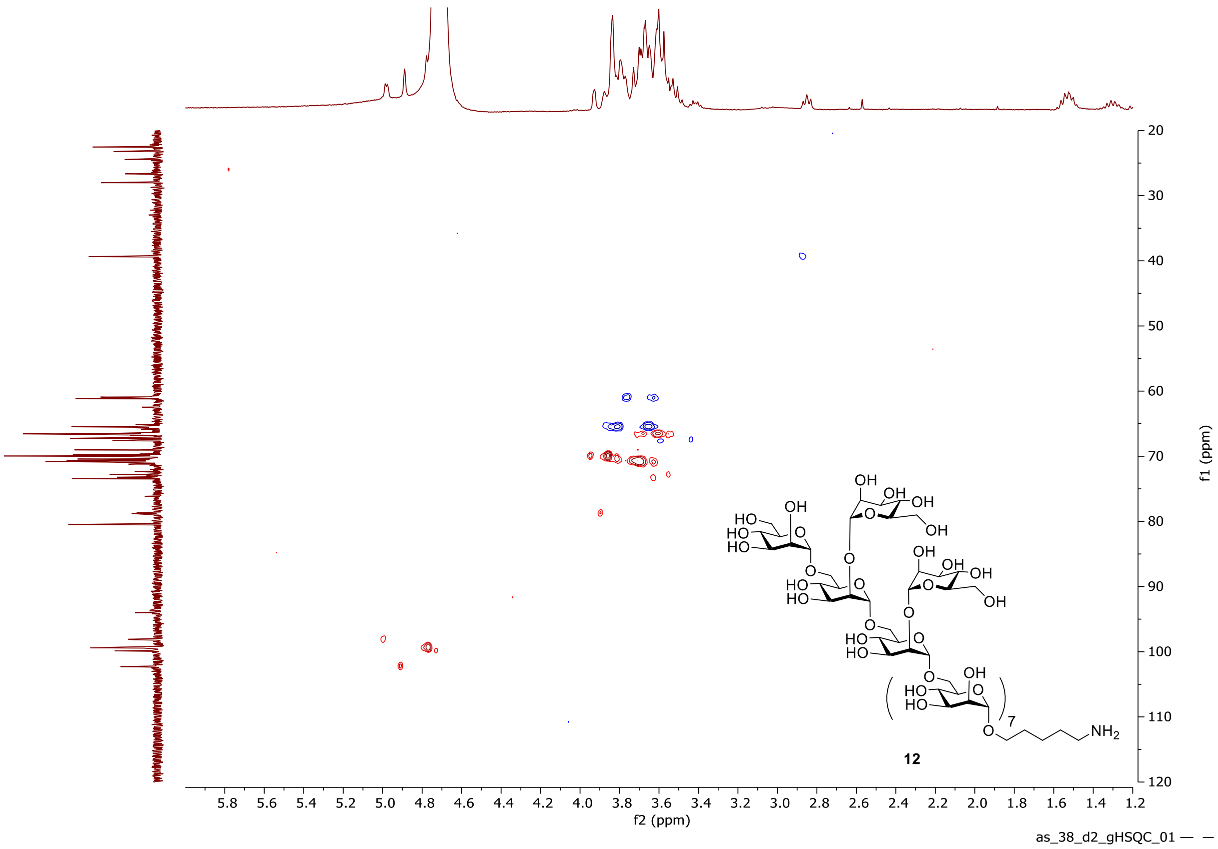

5.4. α(1-6) α(1-5) Linear octaarabinomannoside (**7**)

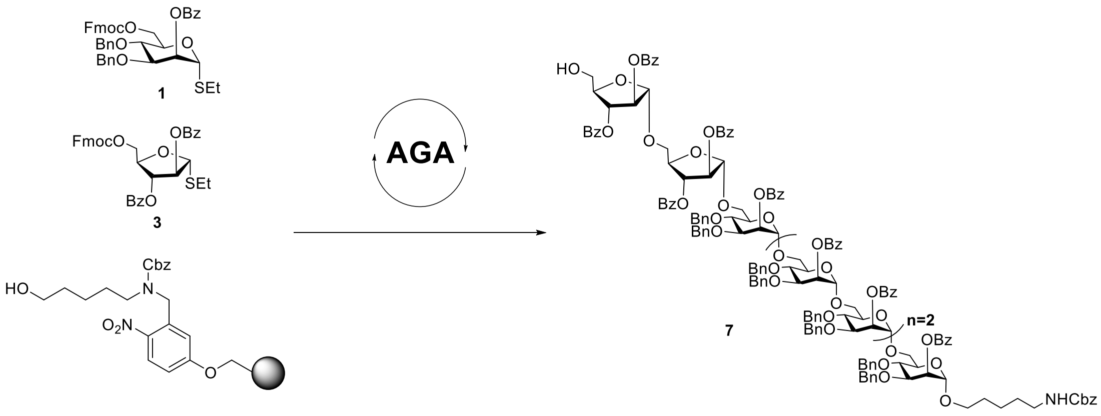

Table S6: Procedure A

	Module	Conditions
	**A**: Resin Preparation for Synthesis	
**6**	**B**: Acidic Wash with TMSOTf Solution<br>**C2**: Thioglycoside Glycosylation<br>**D**: Fmoc Deprotection	**BB1** 6.5 equiv, −40° for 5 min, −20° for 30 min
**2**	**B**: Acidic Wash with TMSOTf Solution<br>**C2**: Thioglycoside Glycosylation<br>**D**: Fmoc Deprotection	**BB3** 13 equiv, −40° for 5 min, −20° for 30 min

Table S7: Procedure B:

	Module	Conditions
	**A**: Resin Preparation for Synthesis	
**6**	**B**: Acidic Wash with TMSOTf Solution<br>**C1**: Thioglycoside Glycosylation<br>**F:** Capping<br>**D**: Fmoc Deprotection	**BB1** 6.5 equiv, −20° for 5 min, 0° for 20 min
**2**	**B**: Acidic Wash with TMSOTf Solution<br>**C1**: Thioglycoside Glycosylation<br>**F:** Capping<br>**D**: Fmoc Deprotection	**BB3** 6.5 equiv, −20° for 5 min, 0° for 20 min

Cleavage from the solid support, as described in Post-synthesizer manipulation, followed by purification using a preparative HPLC (Method B) to provide the fully protected linear octasaccharide **7**. For procedure A: 4 mg, 1.10 µmol, 9%, based on resin loading. For procedure B: 25 mg, 6.8 µmol, 56%, based on resin loading.

Crude NP-HPLC of octasaccharide **7** A) Procedure A. B) Procedure B (ELSD trace, Method A, t_R = 28.6 min).

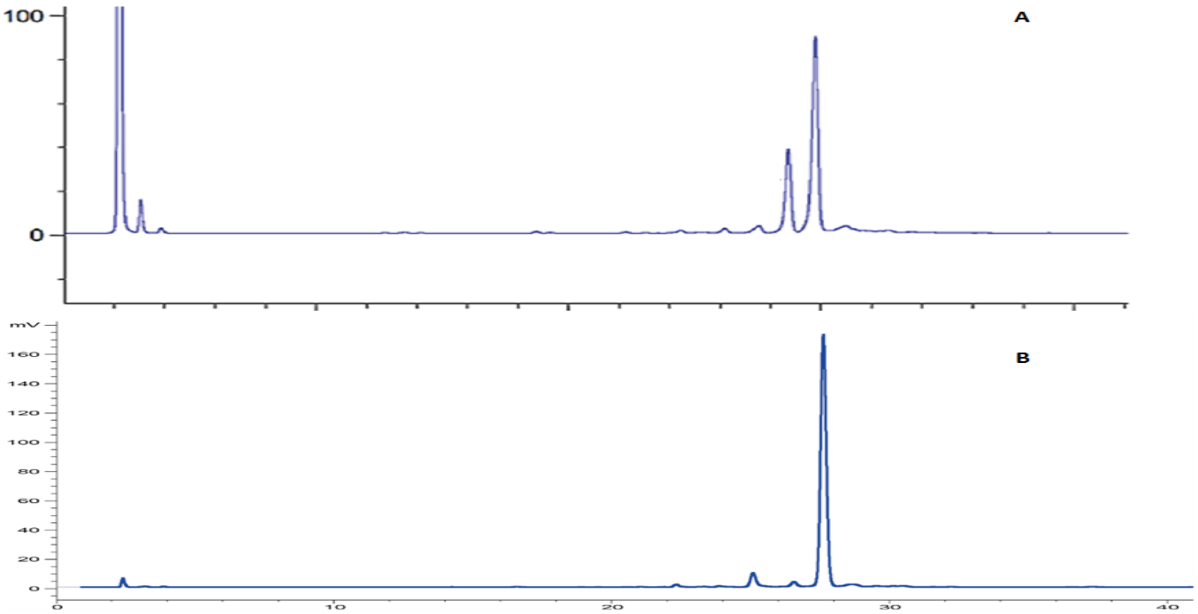

^{1}H NMR (400 MHz, $CDCl_3$) δ 8.19–8.08 (m, 12H), 8.06–8.01 (m, 4H), 7.83–7.76 (m, 4H), 7.62–7.56 (m, 1H), 7.53–7.05 (m, 90H), 6.98–6.89 (m, 4H), 5.88–5.74 (m, 6H), 5.62 (dd, *J* = 3.3, 1.9 Hz, 1H), 5.55 (dd, *J* = 7.9, 2.8 Hz, 2H), 5.42 (s, 1H), 5.37–5.30 (m, 2H), 5.10–5.01 (m, 6H), 4.90–4.75 (m, 12H), 4.66 (d, *J* = 11.4 Hz, 1H), 4.54 (d, *J* = 11.0 Hz, 1H), 4.48–4.31 (m, 12H), 4.26–4.19 (m, 1H), 4.13 (d, *J* = 3.6 Hz, 1H), 4.09–3.99 (m, 8H), 3.99–3.90 (m, 7H), 3.90–3.81 (m, 4H), 3.79–3.70 (m, 6H), 3.67 (d, *J* = 8.6 Hz, 3H), 3.58 (t, *J* = 10.4 Hz, 3H), 3.51–3.35 (m, 5H), 3.19–3.10 (m, 2H), 1.52–1.42 (m, 4H), 1.36–1.32 ppm (m, 2H); ^{13}C NMR (150 MHz, $CDCl_3$) δ 165.95, 165.80, 165.57, 165.47, 165.29, 165.02, 138.54, 138.51, 138.47, 138.45, 138.40, 138.22, 137.90, 137.60, 137.55, 137.49, 137.26, 133.49, 133.46, 133.42, 133.25, 133.21, 133.16, 133.10, 133.06, 129.96, 129.91, 129.82, 129.73, 129.68, 129.59, 129.14, 128.90, 128.59, 128.50, 128.44, 128.38, 128.29, 128.24, 128.17, 128.15, 128.12, 127.99, 127.63, 127.59, 127.37, 127.30, 127.21, 127.07, 127.04, 126.97, 105.99, 105.78, 98.45, 98.42, 98.09, 97.83, 83.50, 83.22, 81.55, 81.44, 78.55, 78.17, 77.97, 75.13, 75.01, 74.97, 74.17, 73.99, 73.87, 73.79, 73.73, 73.69, 71.77, 71.60, 71.54, 71.38, 71.30, 71.25, 71.20, 71.15, 70.96, 70.87, 70.70, 70.04, 69.03, 68.53, 68.41, 68.36, 68.07, 67.76, 67.73, 66.51, 66.08, 65.81, 65.78, 65.75, 65.64, 65.53, 62.19, 40.92, 29.74, 29.00, 23.39 ppm; HRMS (*m*/*z*): $[M + Na]^+$ calcd for $C_{213}H_{207}NO_{51}Na$, 3617.352; found, 3617.345 $[M + Na]^+$.

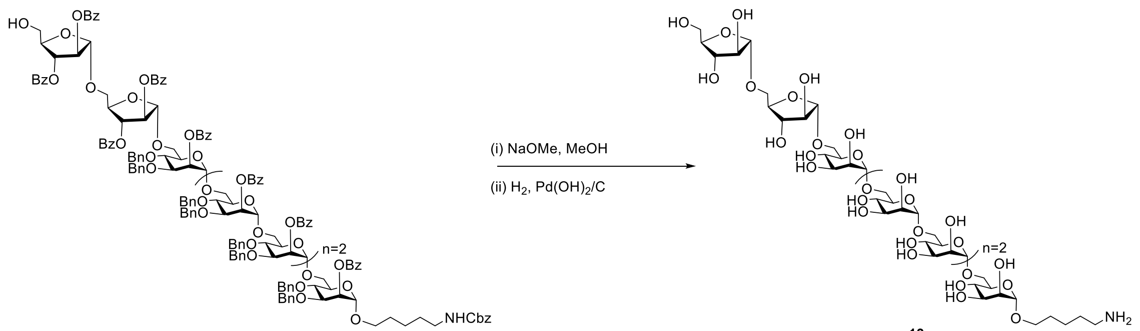

7 **13**

Deprotection of **7** as described in Module G and I, followed by purification using preparative HPLC (Method E), afforded compound **13**. (1.8 mg, 1.34 µmol, 26% over two steps).^{1}H NMR (700 MHz, D_2O) δ 5.12 (s, 1H), 5.11 (s, 1H), 4.94–4.91 (m, 5H), 4.89 (s, 1H), 4.25 (s, 1H), 4.16 (d, *J* = 6.7 Hz, 2H), 4.12 (s, 1H), 4.04 (s, 1H), 4.01 (s, 5H), 3.97 (q, *J* = 11.5, 9.6 Hz, 8H), 3.93–3.72 (m, 28H), 3.67 (dd, *J* = 11.8, 4.5 Hz, 1H), 3.62–3.57 (m, 1H), 3.02 (t, *J* = 7.7 Hz, 3H), 1.74–1.67 (m, 3H), 1.52–1.43 ppm (m, 2H); ^{13}C NMR (151 MHz, D_2O) δ 107.32, 107.18, 99.82, 99.35, 99.31, 99.26, 83.87, 82.03, 80.83, 80.76, 76.59, 76.44, 70.90, 70.85, 70.76, 70.62, 70.49, 69.99, 69.91, 69.86, 67.55, 66.68, 66.57, 66.52, 65.44, 61.10, 39.30, 27.96, 26.51, 22.47 ppm. HRMS (*m*/*z*): $[M + H]^+$ calcd for $C_{51}H_{89}NO_{39}$, 1340.508; found, 1340.501.

RP-HPLC of purified hexasaccharide **13** (ELSD trace, Method C, t_R = 25.5 min).

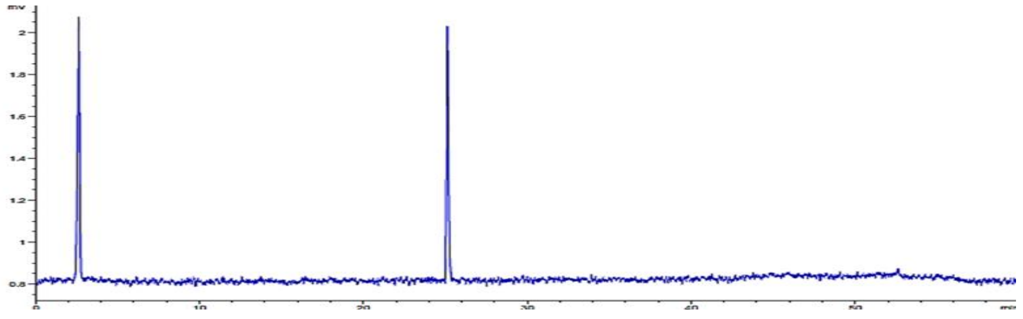

^{1}H NMR of 13 (700 MHz, D_2O)

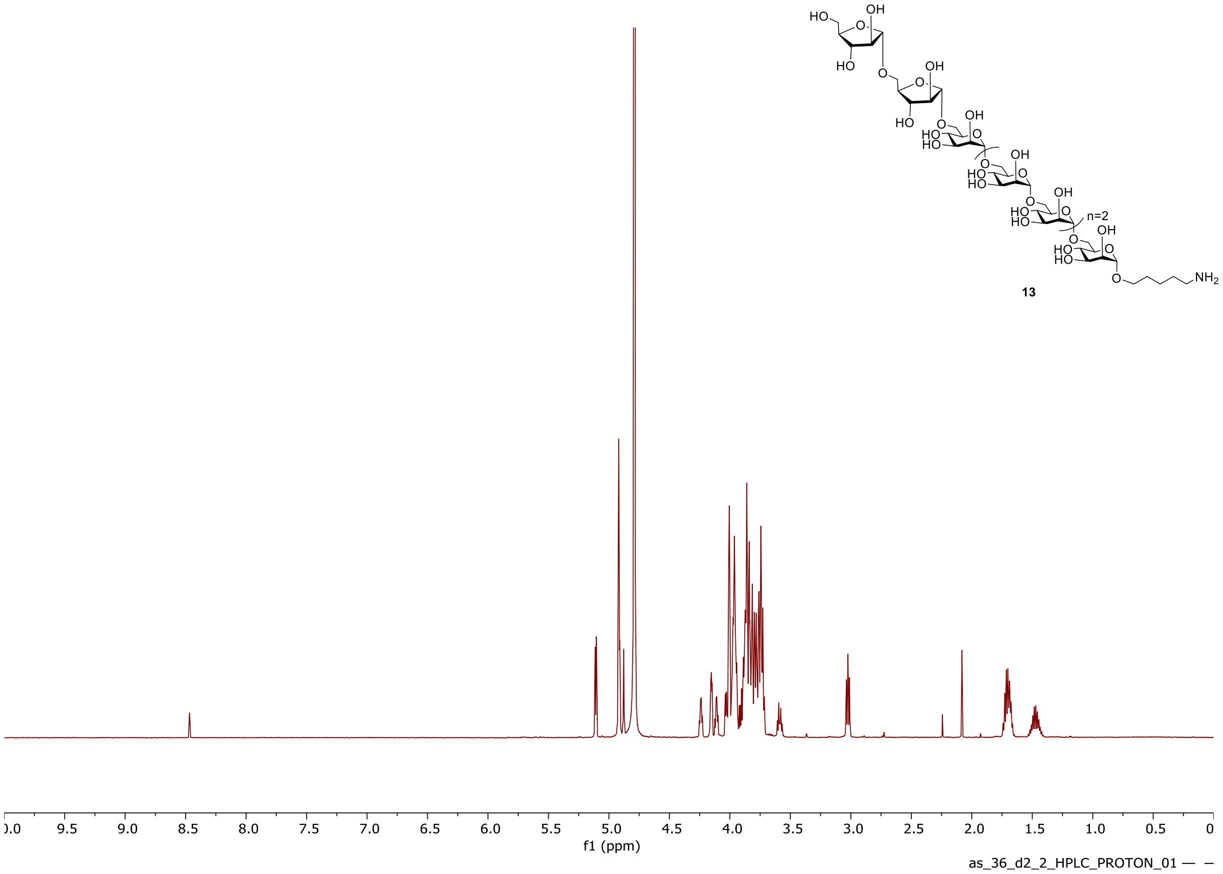

^{13}C NMR of 13 (150 MHz, D_2O)

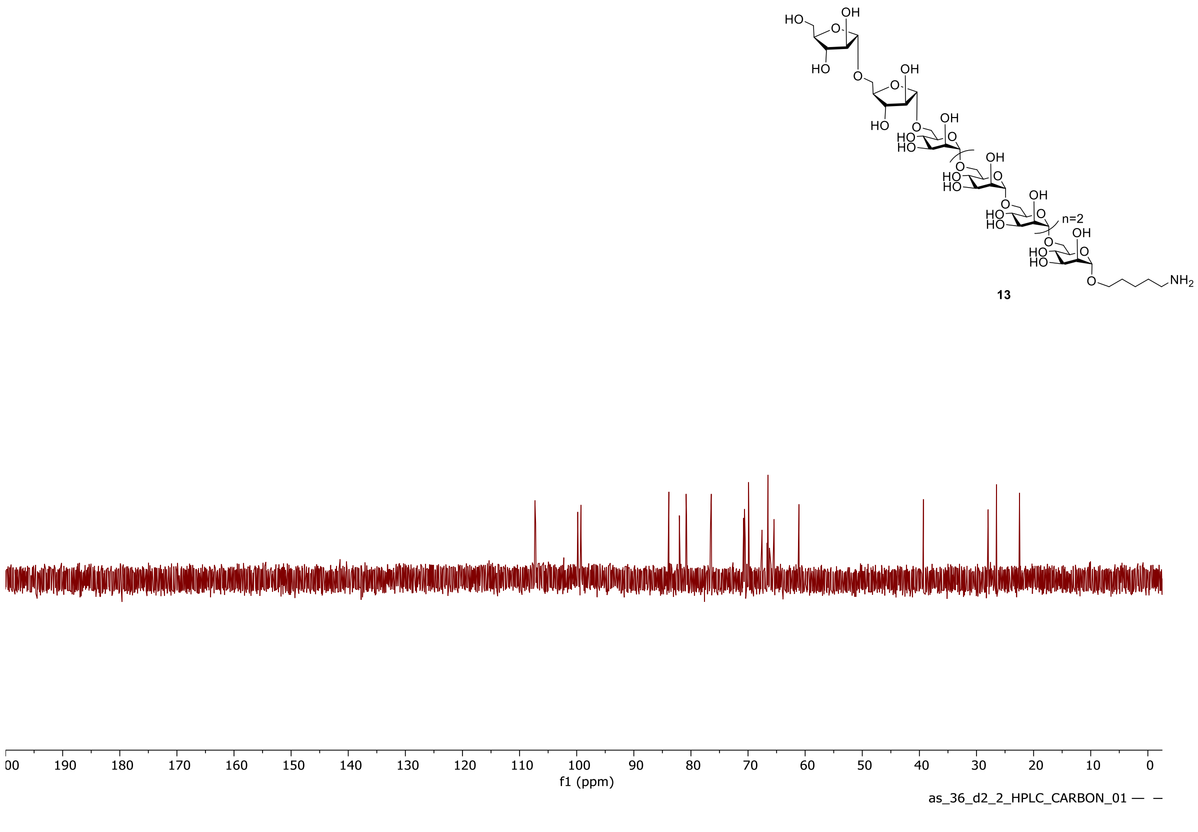

HSQC NMR of 13 (D_2O)

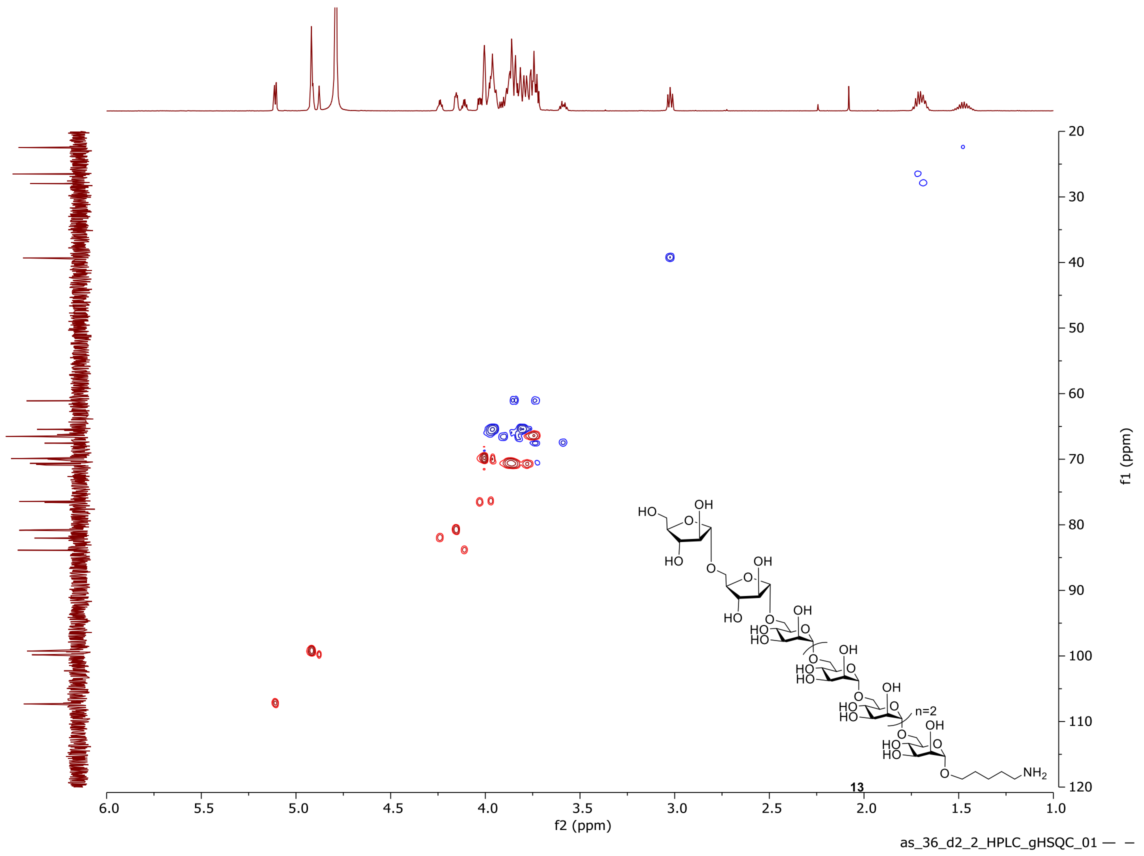

5.5. α(1-6) α(1-5) linear dodecaarabinomannoside (**8**)

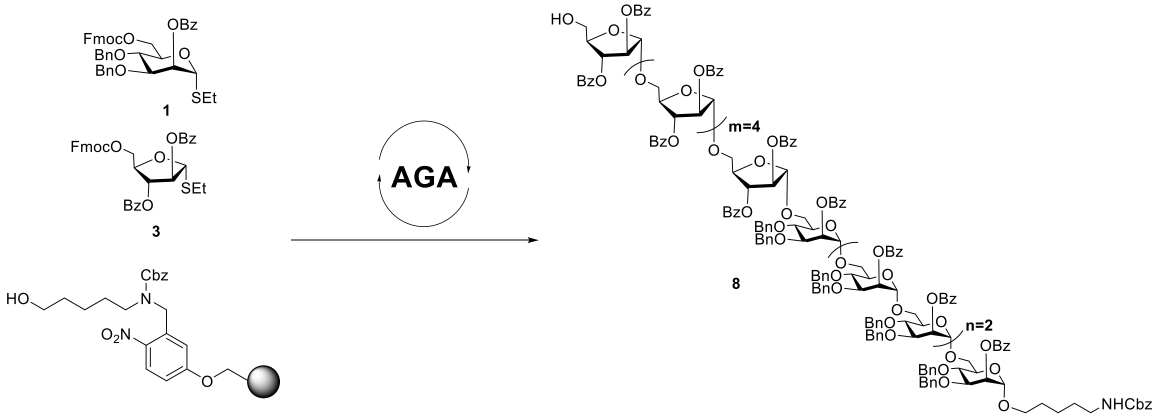

Table S8: Procedure A

	Module	Conditions
	**A**: Resin Preparation for Synthesis	
6	**B**: Acidic Wash with TMSOTf Solution<br>**C2**: Thioglycoside Glycosylation<br>**D**: Fmoc Deprotection	**BB1** 6.5 equiv, −40° for 5 min, −20° for 30 min
6	**B**: Acidic Wash with TMSOTf Solution<br>**C2**: Thioglycoside Glycosylation<br>**D**: Fmoc Deprotection	**BB3** 13 equiv, −40° for 5 min, −20° for 30 min

Table S9: Procedure B:

	Module	Conditions
	**A**: Resin Preparation for Synthesis	
6	**B**: Acidic Wash with TMSOTf Solution<br>**C1**: Thioglycoside Glycosylation<br>**F:** Capping<br>**D**: Fmoc Deprotection	**BB1** 6.5 equiv, −20° for 5 min, 0° for 20 min
6	**B**: Acidic Wash with TMSOTf Solution<br>**C1**: Thioglycoside Glycosylation<br>**F:** Capping<br>**D**: Fmoc Deprotection	**BB3** 6.5 equiv, −20° for 5 min, 0° for 20 min

Cleavage from the solid support, as described in Post-synthesizer manipulations, followed by purification using a preparative HPLC (Method B) to provide the fully protected linear dodecasaccharide **8**. For procedure A: 4 mg, 0.80 μmol, 7%, based on resin loading. For Procedure B: 38 mg, 7.38 μmol, 61%, based on resin loading.

Crude NP-HPLC of dodecasaccharide **8**: A) Procedure A. B) Procedure B (ELSD trace, Method A, t_R = 36.5 min).

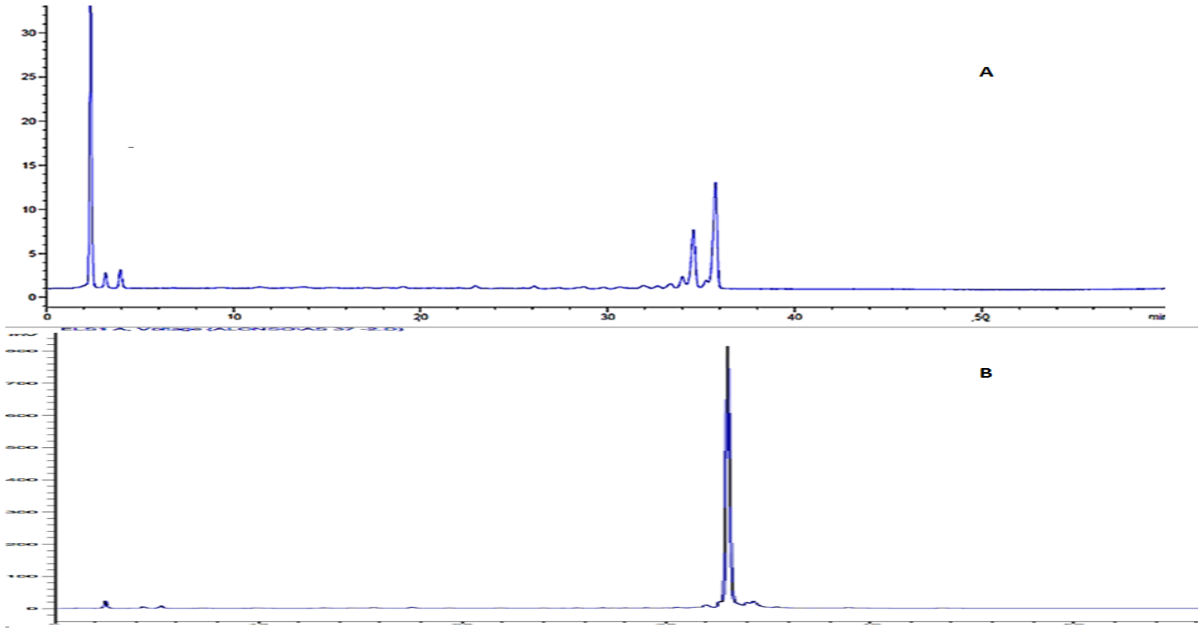

^{1}H NMR (600 MHz, $CDCl_3$) δ 8.17–7.98 (m, 24H), 7.90–7.75 (m, 12H), 7.61–7.05 (m, 115H), 6.98–6.88 (m, 4H), 5.86–5.74 (m, 6H), 5.63–5.54 (m, 11H), 5.41–5.34 (m, 6H), 5.31 (s, 1H), 5.15–4.99 (m, 7H), 4.89–4.74 (m, 11H), 4.66 (d, *J* = 11.4 Hz, 1H), 4.58–4.51 (m, 3H), 4.48–4.21 (m, 10H), 4.15–3.37 (m, 50H), 3.20–3.10 ppm (m, 2H); ^{13}C NMR (100 MHz, $CDCl_3$) δ 165.76, 165.66, 165.60, 165.22, 138.64, 138.59, 137.78, 137.68, 133.64, 133.54, 133.44, 133.21, 130.15, 129.96, 129.89, 129.76, 129.26, 129.14, 128.78, 128.63, 128.47, 128.35, 127.82, 127.77, 127.37, 127.24, 127.12, 106.01, 98.64, 98.27, 83.76, 83.48, 82.19, 81.64, 81.59, 75.32, 75.20, 71.48, 71.08, 69.22, 68.61, 66.72, 66.25, 65.96, 29.86, 29.19 ppm; HRMS (*m*/*z*): $[M + Na]^+$ calcd for $C_{161}H_{168}NO_{37}Na$, 4979.738; found, 4979.837.

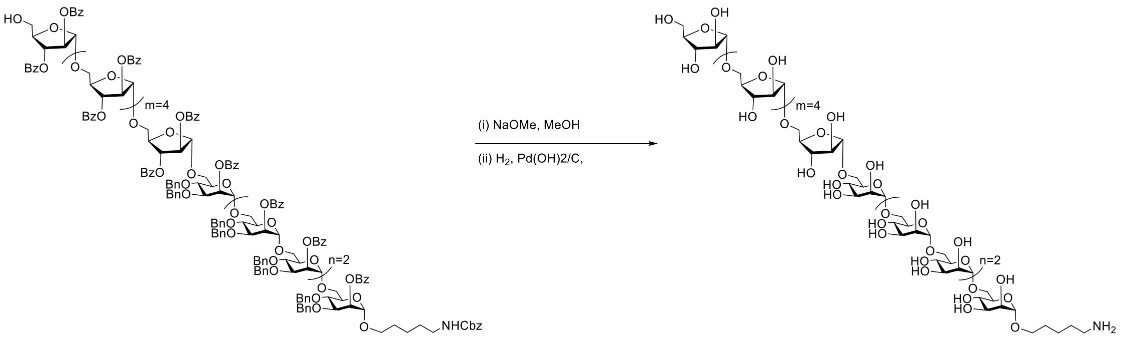

8 14

Deprotection of **8**, as described in Modules G and I, followed by purification using preparative HPLC (Method E) afforded compound **14** (3.6 mg, 1.90 µmol, 25% over two steps). ^{1}H NMR (700 MHz, D_2O) δ 5.13–5.11 (m, 6H), 4.94–4.91 (m, 5H), 4.89 (s,

1H), 4.26–4.22 (m, 5H), 4.17–4.15 (m, 6H), 4.13–4.10 (m, 1H), 4.06–3.94 (m, 17H), 3.94–3.72 (m, 37H), 3.67 (dd, J = 11.6, 4.3 Hz, 1H), 3.61–3.57 (m, 1H), 3.02 (t, J = 7.7 Hz, 2H), 1.74–1.66 (m, 4H), 1.53–1.43 ppm (m, 2H); ^{13}C NMR (176 MHz, D_2O) δ 107.47, 107.37, 107.25, 99.87, 99.39, 99.35, 99.30, 99.27, 83.92, 82.31, 82.30, 82.06, 80.87, 80.80, 80.77, 76.68, 76.63, 76.47, 70.94, 70.90, 70.88, 70.81, 70.78, 70.76, 70.69, 70.65, 70.53, 70.03, 69.95, 69.90, 67.59, 66.83, 66.72, 66.60, 66.56, 66.54, 66.52, 66.29, 65.63, 65.52, 65.47, 61.13, 46.65, 39.33, 28.01, 26.54, 22.51 ppm. HRMS (m/z): $[M + H]^+$ calcd for $C_{71}H_{121}NO_{55}$, 1868.677; found, 1868.680.

For the arabinomannanosides **14**, the acid labile arabinose chain was cleaved during hydrogenation, giving a complex mixture of deletion sequences lacking one to six arabinose moieties.

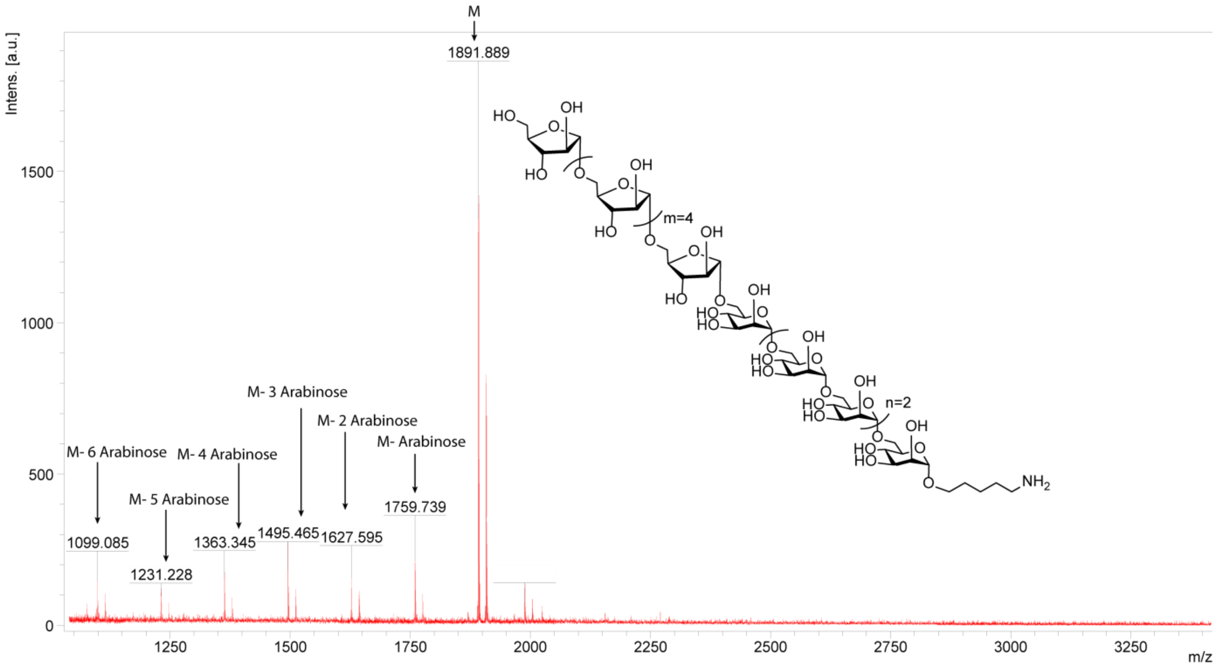

Hydrogenolysis with $Pd(OH)_2$ was performed to access the fully deprotected **14**.

RP-HPLC of purified dodecasaccharide **14** (ELSD trace, Method C, t_R = 29.6 min)

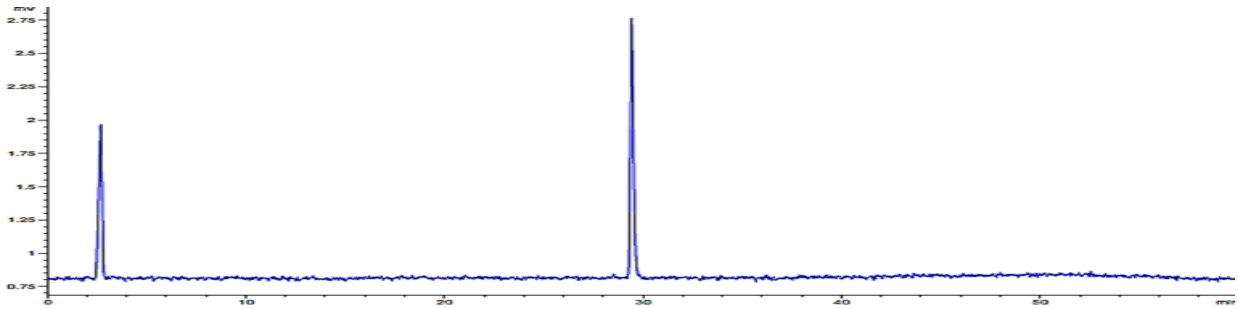

^{1}H NMR of 14 (700 MHz, D_2O)

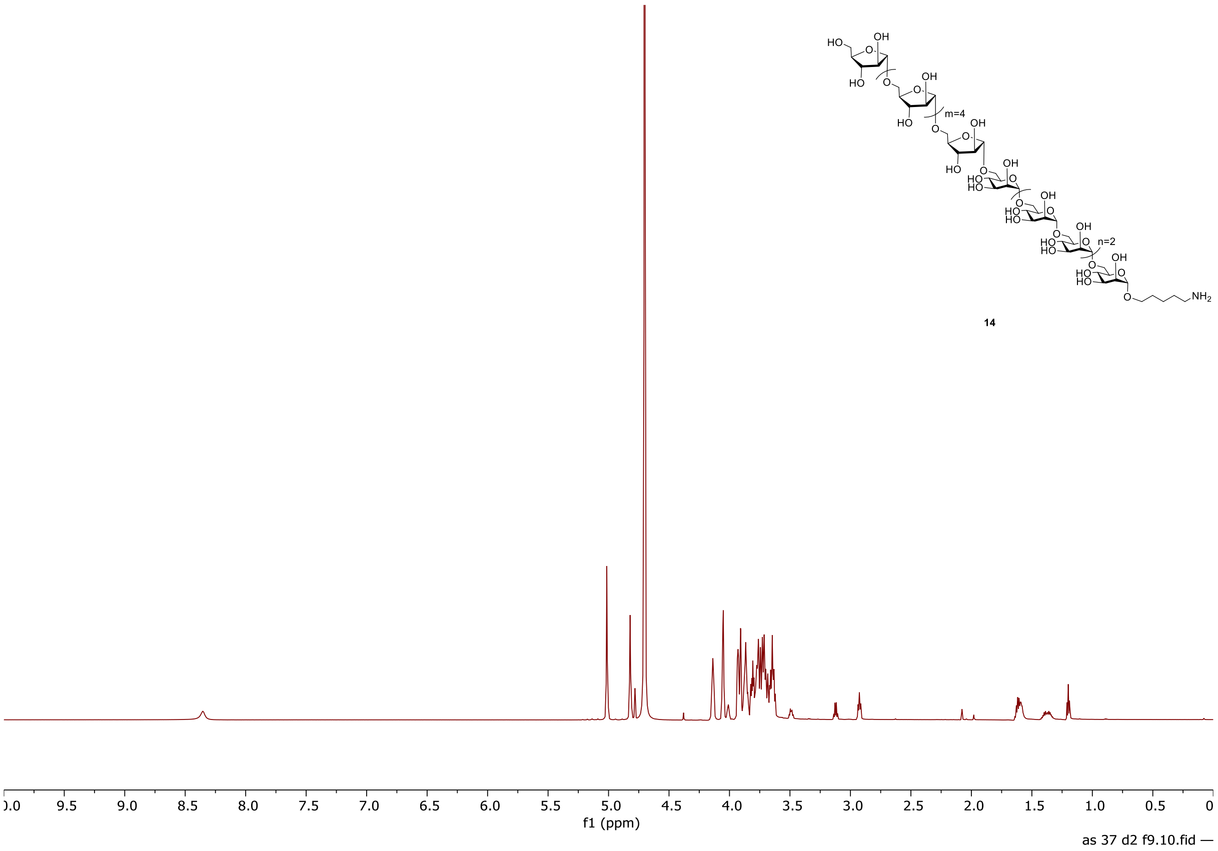

^{13}C NMR of 14 (150 MHz, D_2O)

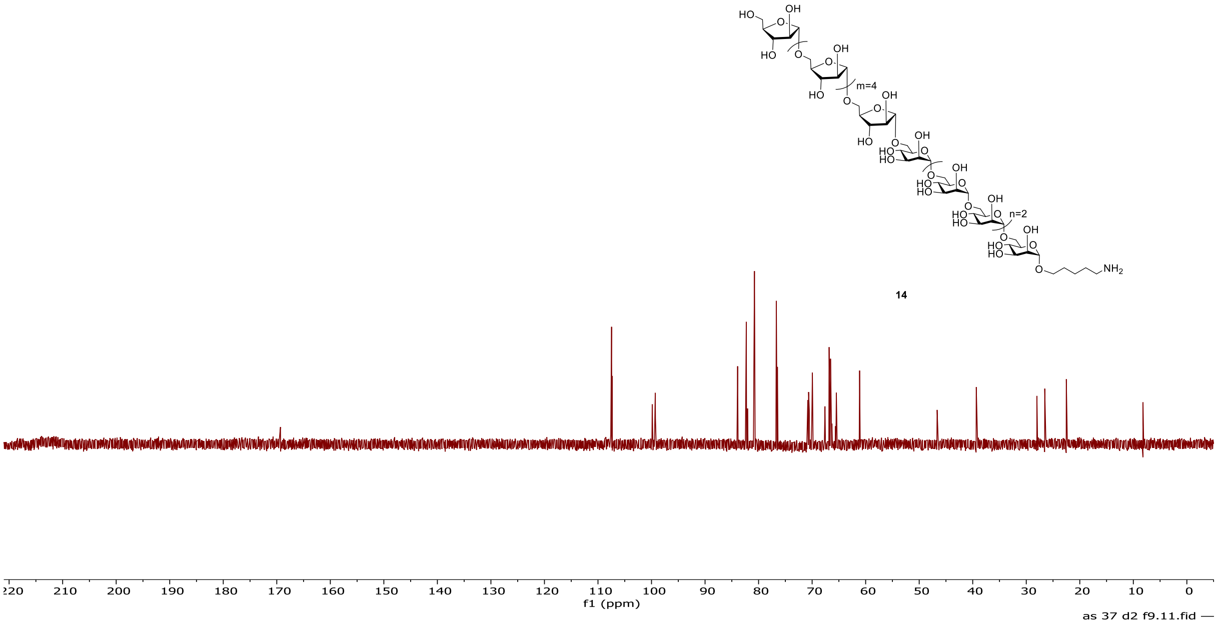

HSQC NMR of 14 (D_2O)

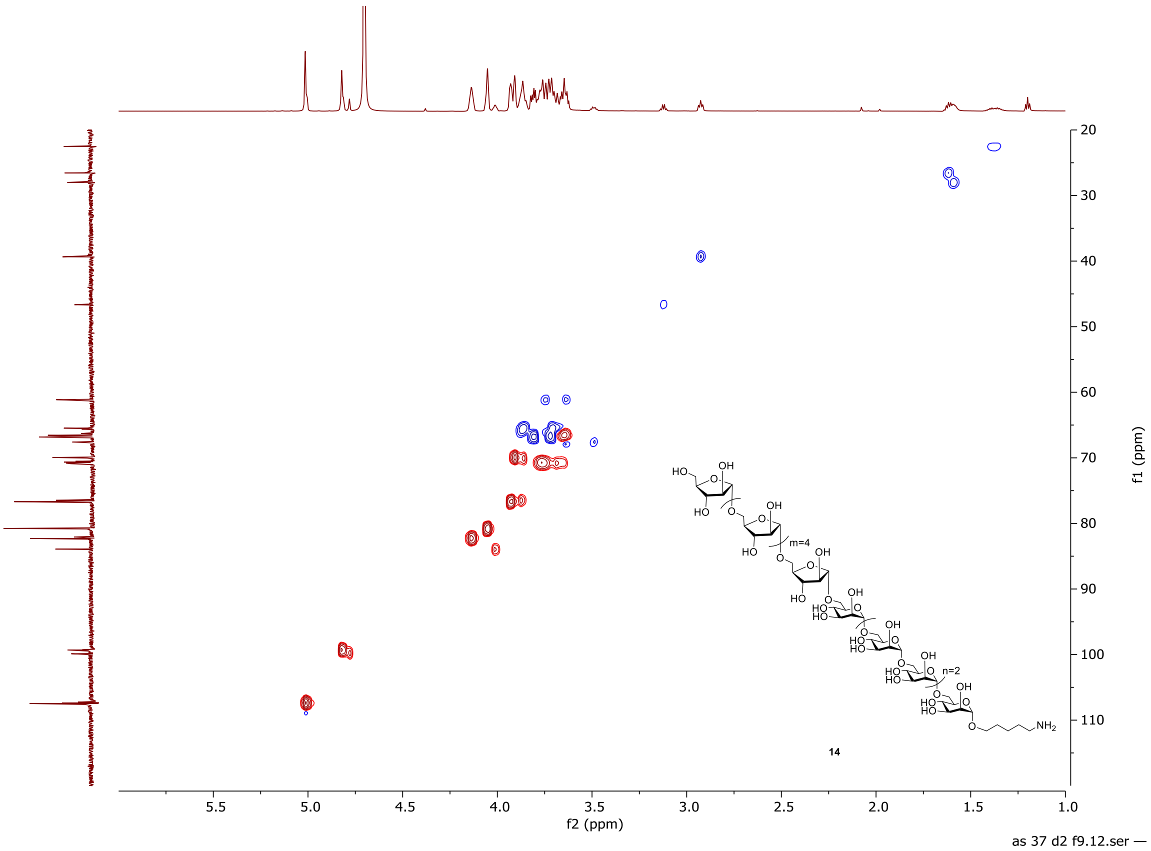

5.6. α(1-6) α(1-2) α(1-5) Branched dodecaarabinomannoside (**9**)

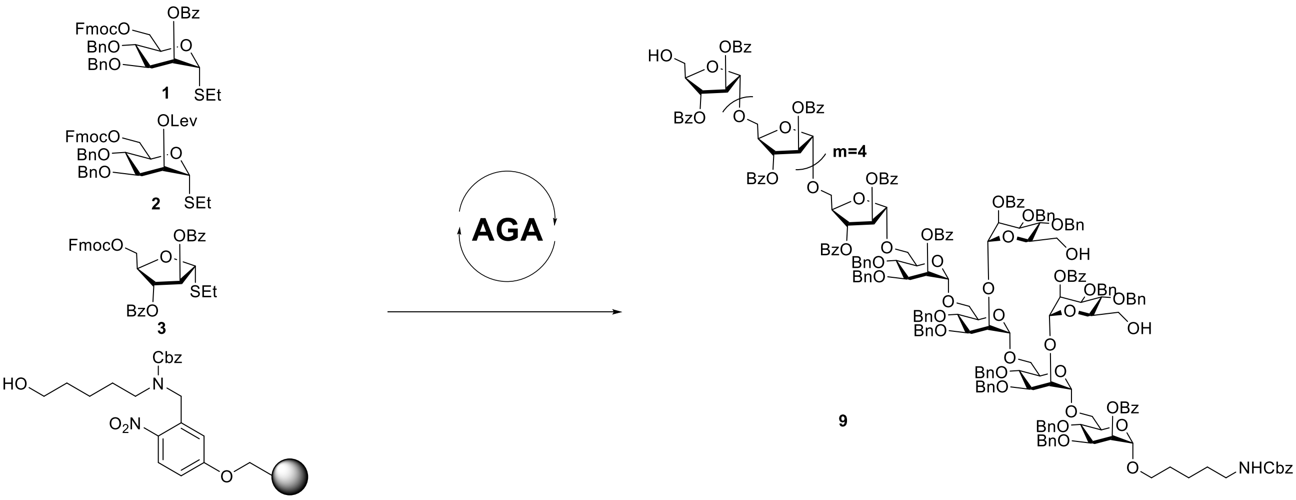

Table S10: Procedure A

	Module	Conditions
	A: Resin Preparation for Synthesis	
	B: Acidic Wash with TMSOTf Solution<br>C2: Thioglycoside Glycosylation<br>D: Fmoc Deprotection	BB **1** 6.5 equiv, −40° for 5 min, −20° for30 min
**2**	B: Acidic Wash with TMSOTf Solution<br>C2: Thioglycoside Glycosylation<br>D: Fmoc Deprotection	BB **2** 6.5 equiv, −40° for 5 min, −20° for 30 min
	B: Acidic Wash with TMSOTf Solution<br>C2: Thioglycoside Glycosylation<br>D: Fmoc Deprotection	BB **1** 6.5 equiv, −40° for 5 min, −20° for 30 min
**6**	B: Acidic Wash with TMSOTf Solution<br>C2: Thioglycoside Glycosylation<br>D: Fmoc Deprotection	BB **3** 13 equiv, −40° for 5 min, −20° for 30 min
	E: Lev Deprotection<br>B: Acidic Wash with TMSOTf Solution<br>2 × C2: Thioglycoside Glycosylation<br>D: Fmoc Deprotection	BB **1** 13 equiv, −40° for 5 min, −20° for 30 min

Table S11: Procedure B

	Module	Conditions
	A: Resin Preparation for Synthesis	
	B: Acidic Wash with TMSOTf Solution<br>C1: Thioglycoside Glycosylation<br>F: Capping<br>D: Fmoc Deprotection	BB **1** 6.5 equiv, −20° for 5 min, 0° for 20 min
**2**	B: Acidic Wash with TMSOTf Solution<br>C1: Thioglycoside Glycosylation<br>F: Capping<br>D: Fmoc Deprotection	BB **2** 6.5 equiv, −20° for 5 min, 0° for 20 min
	B: Acidic Wash with TMSOTf Solution<br>C1: Thioglycoside Glycosylation<br>F: Capping<br>D: Fmoc Deprotection	BB **1** 6.5 equiv, −20° for 5 min, 0° for 20 min
**6**	B: Acidic Wash with TMSOTf Solution<br>C1: Thioglycoside Glycosylation<br>F: Capping<br>D: Fmoc Deprotection	BB **3** 6.5 equiv, −20° for 5 min, 0° for 20 min
	E: Lev Deprotection<br>B: Acidic Wash with TMSOTf Solution<br>2 × C1: Thioglycoside Glycosylation<br>F: Capping<br>D: Fmoc Deprotection	BB **1** 6.5 equiv, −20° for 5 min, 0° for 20 min

Cleavage from the solid support, as described in Post-synthesizer manipulations, followed by purification using a preparative HPLC (Method B, t_R = 38.8 min), to provide the fully protected branched dodecasaccharide **9**. For procedure A: 2 mg, 0.41 µmol, 3%, based on resin loading. For procedure B: 16 mg, 3.22 µmol, 26%, based on resin loading.

Crude NP-HPLC of dodecasaccharide 9. A) Procedure A. B) Procedure B (ELSD trace, Method A, t_R = 38.8 min).

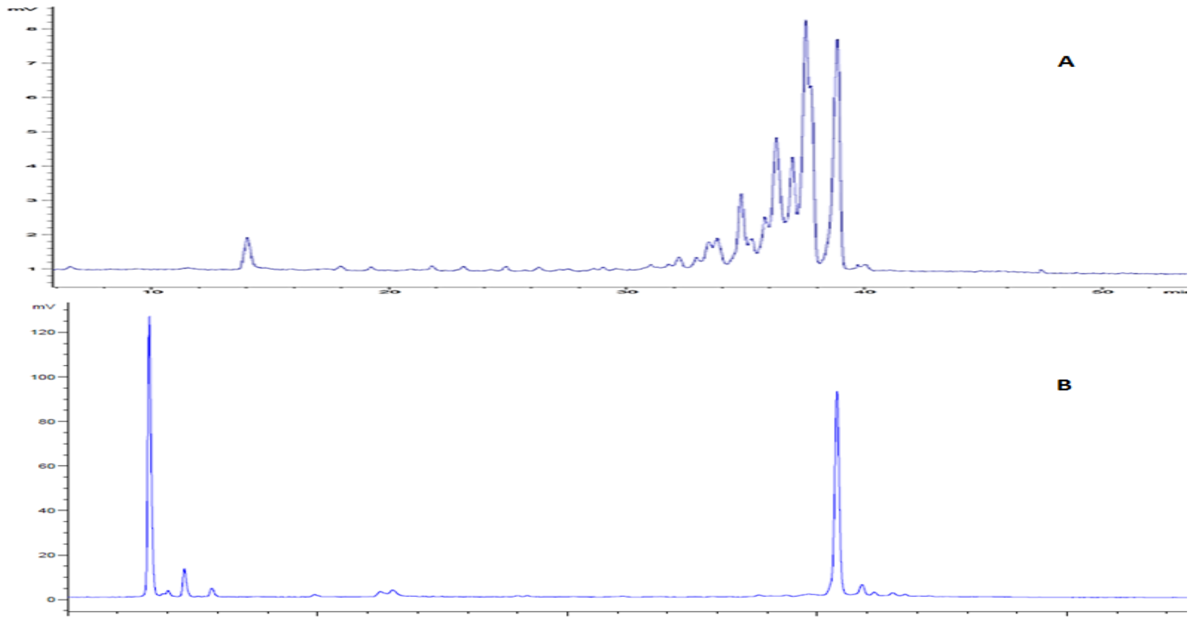

^{1}H NMR (600 MHz, chloroform-*d*) δ 8.16–8.07 (m, 3H), 8.05–7.98 (m, 25H), 7.96–7.92 (m, 2H), 7.87 (dd, *J* = 8.2, 1.4 Hz, 4H), 7.82 (ddd, *J* = 8.3, 6.8, 1.4 Hz, 12H), 7.76 (dt, *J* = 8.4, 1.7 Hz, 7H), 5.88 (d, *J* = 2.4 Hz, 1H), 5.74 (s, 1H), 5.69–5.64 (m, 1H), 5.64–5.54 (m, 19H), 5.16 (d, *J* = 1.7 Hz, 1H), 5.05 (d, *J* = 5.7 Hz, 6H), 4.97 (d, *J* = 1.9 Hz, 1H), 4.92–4.33 (m, 57H), 4.27–3.59 (m, 61H), 3.58–3.31 (m, 8H), 3.13 (q, *J* = 6.8 Hz, 4H), 1.56–1.50 (m, 4H), 1.46 (t, *J* = 7.5 Hz, 2H), 1.34–1.29 ppm (m, 2H). ^{13}C NMR (151 MHz, $CDCl_3$) δ 166.02, 165.58, 165.55, 165.52, 165.42, 165.33, 165.05, 165.02, 138.62, 138.37, 138.07, 137.99, 137.91, 137.79, 136.64, 133.45, 133.35, 133.28, 133.24, 133.09, 133.06, 133.01, 129.87, 129.82, 129.78, 129.70, 129.67, 129.61, 129.57, 129.18, 129.10, 129.00, 128.92, 128.63, 128.54, 128.47, 128.45, 128.43, 128.36, 128.34, 128.25, 128.24, 128.22, 128.19, 128.17, 128.14, 128.11, 128.07, 128.04, 127.99, 127.96, 127.94, 127.83, 127.74, 127.71, 127.69, 127.59, 127.49, 127.41, 127.32, 127.26, 127.14, 126.90, 106.00, 105.83, 105.80, 99.34, 97.52, 83.58, 83.29, 82.02, 81.91, 81.63, 81.53, 81.46, 78.37, 77.96, 77.65, 75.13, 74.92, 74.83, 74.05, 73.26, 71.79, 71.66, 71.59, 71.26, 71.16, 70.64, 66.51, 66.08, 65.79, 65.72, 65.61, 62.45, 62.28, 40.91, 29.72, 28.97, 23.39 ppm. HRMS (*m*/*z*): $[2M + Na]^+$ calcd for $C_{275}H_{263}NO_{73}Na$, 2397.337; found, 2397.338).

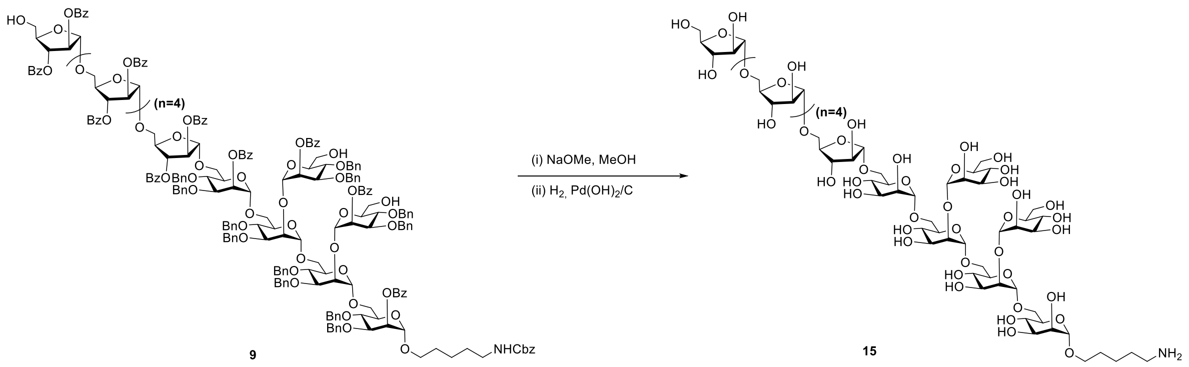

Deprotection of **9**, as described in Modules G and I, followed by purification using preparative HPLC (Method E) afforded compound **15** (1.0 mg, 0.53 µmol, 17% over two steps). ^{1}H NMR (700 MHz, D_2O) δ 5.16 (s, 1H), 5.15 (s, 1H), 5.12 (s, 6H), 5.07 (s, 1H), 5.06 (s, 1H), 4.95 (s, 1H), 4.88 (s, 1H), 4.24 (s, 5H), 4.17 (d, *J* = 10.3 Hz, 6H), 4.11 (s, 3H), 4.07–3.94 (m, 15H), 3.94–3.90 (m, 7H), 3.87–3.81 (m, 15H), 3.80–3.66 (m, 16H), 3.61–3.57 (m, 1H), 3.03 (t, *J* = 7.6 Hz, 2H), 1.74–1.67 (m, 4H), 1.52–1.44 ppm (m, 2H); ^{13}C NMR (176 MHz, D_2O) δ 107.5, 107.4, 102.3, 99.9, 99.6, 98.1, 84.0, 82.3, 82.1, 80.9, 80.8, 80.8, 78.8, 76.7, 76.7, 76.7, 76.5, 73.3, 73.2, 71.2, 70.9, 70.9, 70.7, 70.5, 70.5, 70.1, 70.0, 70.0, 67.6, 66.9, 66.9, 66.8, 66.7, 66.7, 66.6, 66.5, 66.5, 66.3, 65.9, 61.2, 61.1, 61.0, 43.8, 39.4, 28.0, 26.6, 22.5 ppm. HRMS (*m*/*z*): $[M + H]^+$ calcd for $C_{71}H_{121}NO_{55}$, 1868.677; found, 1868.669.

RP-HPLC of purified dodecasaccharide **15** (ELSD trace, Method C, t_R = 28.5 min).

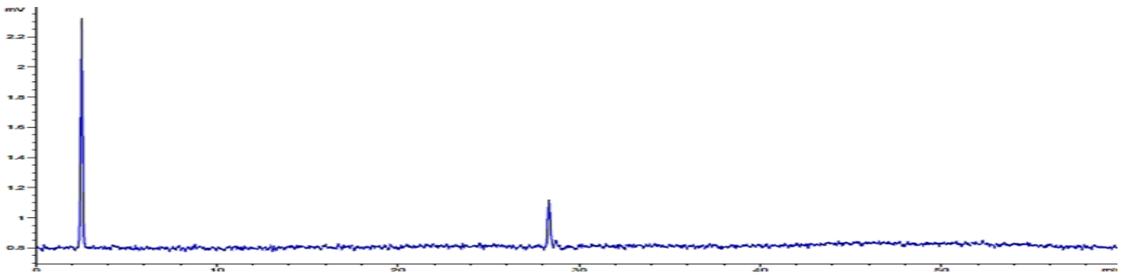

^{1}H NMR of 15 (700 MHz, D_2O)

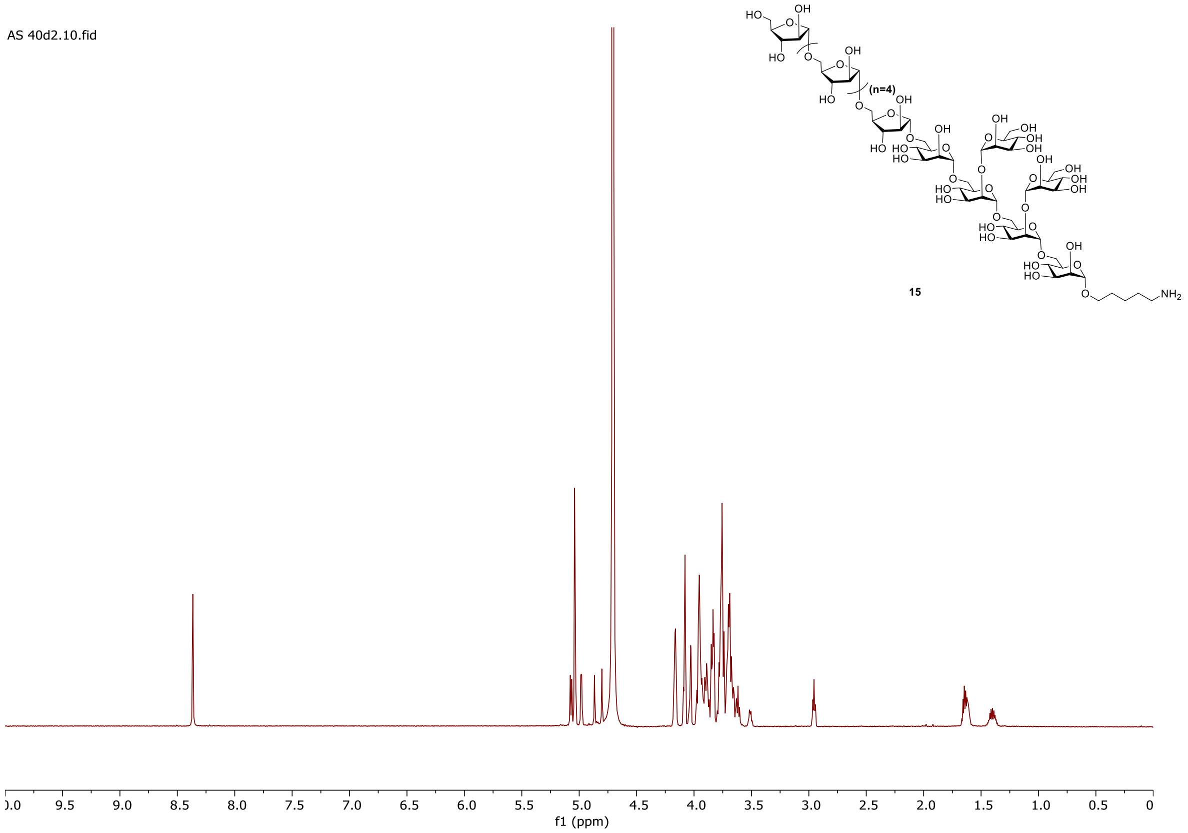

^{13}C NMR of 15 (150 MHz, D_2O)

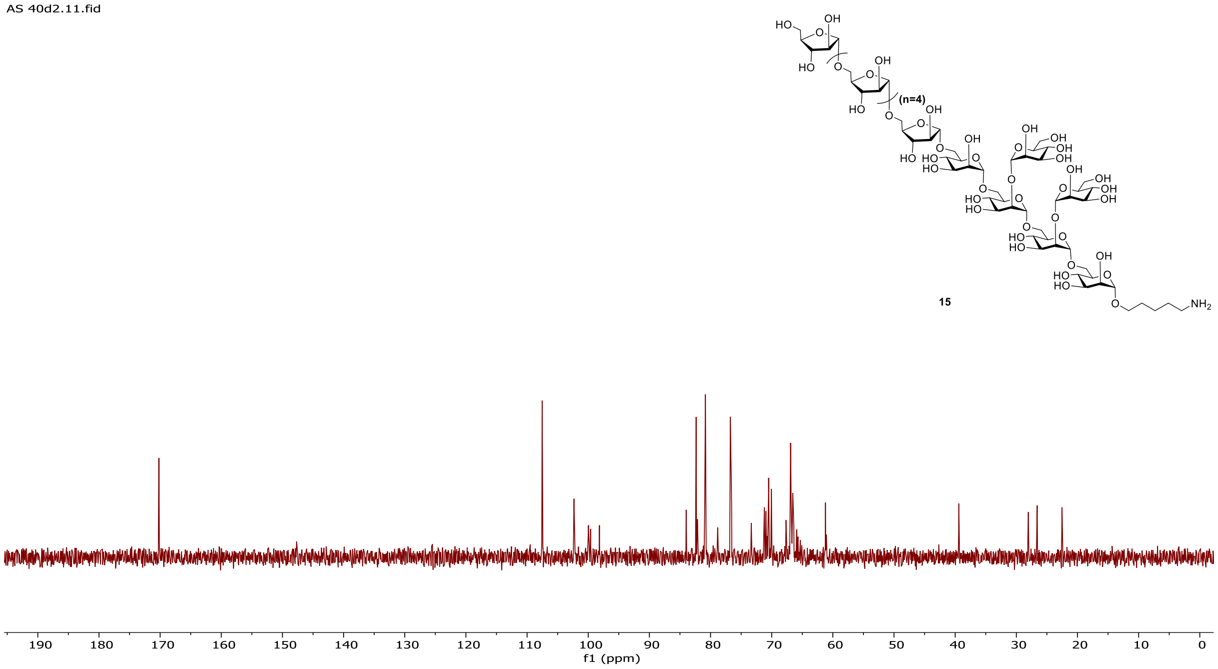

HSQC NMR of 15 (D_2O)

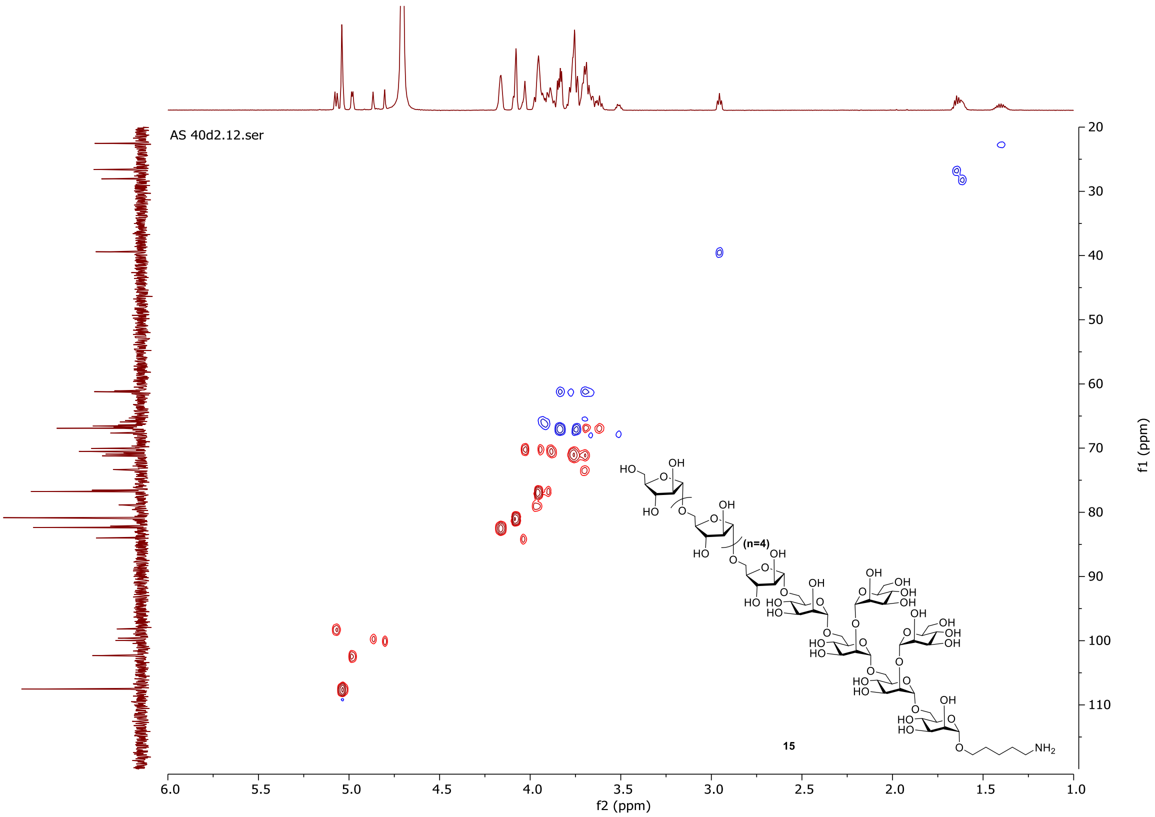

6. References

1. Hashimoto, Y.; Tanikawa, S.; Saito, R.; Sasaki, K. *J. Am. Chem. Soc.* **2016**, *138* (45), 14840-14843. doi:10.1021/jacs.6b08874
2. Krock, L.; Esposito, D.; Castagner, B.; Wang, C.-C.; Bindschadler, P.; Seeberger, P. H. *Chem. Sci.* **2012**, *3* (5), 1617-1622, doi:10.1039/c2sc00940d
3. Eller, S.; Collot, M.; Yin, J.; Hahm, H. S.; Seeberger, P. H. *Angew. Chem., Int. Ed.* **2013**, *52* (22), 5858-5861. doi:10.1002/anie.201210132
4. Naresh, K.; Schumacher, F.; Hahm, H. S.; Seeberger, P. H. *Chem. Commun.* **2017**, *53* (65), 9085-9088. doi:10.1039/C7CC04380E
5. Kandasamy, J.; Hurevich, M.; Seeberger, P. H. *Chem. Commun.* **2013**, *49* (40), 4453-4455. doi:10.1039/c3cc00042g
6. Yu, Y.; Kononov, A.; Delbianco, M.; Seeberger, P. H. *Chem. – Eur. J.* **2018**, *24* (23), 6075-6078. doi:10.1002/chem.201801023
7. Eller, S.; Collot, M.; Yin, J.; Hahm, H. S.; Seeberger, P. H. *Angew. Chem., Int. Ed.* **2013**, *52* (22), 5858-5861. doi:10.1002/anie.201210132
